# Unique Gene Expression Signatures in the Intestinal Mucosa and Organoids Derived from Germ-Free and Monoassociated Mice

**DOI:** 10.3390/ijms20071581

**Published:** 2019-03-29

**Authors:** Lucie Janeckova, Klara Kostovcikova, Jiri Svec, Monika Stastna, Hynek Strnad, Michal Kolar, Tomas Hudcovic, Jitka Stancikova, Jolana Tureckova, Nikol Baloghova, Eva Sloncova, Katerina Galuskova, Helena Tlaskalova-Hogenova, Vladimir Korinek

**Affiliations:** 1 Institute of Molecular Genetics, Academy of Sciences of the Czech Republic, Videnska 1083, 142 20 Prague 4, Czech Republic; lucie.janeckova@img.cas.cz (L.J.); klimesov@biomed.cas.cz (K.K.); jiri.svec@img.cas.cz (J.S.); monika.stastna@img.cas.cz (M.S.); hynek.strnad@img.cas.cz (H.S.); kolarmi@img.cas.cz (M.K.); jitka.stancikova@lfmotol.cuni.cz (J.S.); jolana.tureckova@img.cas.cz (J.T.); nikol.baloghova@img.cas.cz (N.B.); slon@img.cas.cz (E.S.); katerina.galuskova@img.cas.cz (K.G.); 2 Institute of Microbiology, Academy of Sciences of the Czech Republic, Videnska 1083, 142 20 Prague 4, Czech Republic; hudcovic@biomed.cas.cz (T.H.); tlaskalo@biomed.cas.cz (H.T.-H.); 3 Department of Radiotherapy and Oncology, Third Faculty of Medicine, Charles University, Prague, Srobarova 50, 100 34 Prague 10, Czech Republic

**Keywords:** expression profiling, Enricher tool, microbiota, monoassociation, Onecut2

## Abstract

Commensal microbiota contribute to gut homeostasis by inducing transcription of mucosal genes. Analysis of the impact of various microbiota on intestinal tissue provides an important insight into the function of this organ. We used cDNA microarrays to determine the gene expression signature of mucosa isolated from the small intestine and colon of germ-free (GF) mice and animals monoassociated with two *E. coli* strains. The results were compared to the expression data obtained in conventionally reared (CR) mice. In addition, we analyzed gene expression in colon organoids derived from CR, GF, and monoassociated animals. The analysis revealed that the complete absence of intestinal microbiota mainly affected the mucosal immune system, which was not restored upon monoassociation. The most important expression changes observed in the colon mucosa indicated alterations in adipose tissue and lipid metabolism. In the comparison of differentially expressed genes in the mucosa or organoids obtained from GF and CR mice, only six genes were common for both types of samples. The results show that the increased expression of the angiopoietin-like 4 (*Angptl4*) gene encoding a secreted regulator of lipid metabolism indicates the GF status.

## 1. Introduction

Mammalian gut is a complex organ consisting of cells and tissues intrinsic for the organism, and in addition, containing vast amounts of bacteria. The microbial population in the gut, so-called microbiome, outnumbers the amount of cells present in the human body (reviewed in Reference [1]). The presence of the commensal microbes is required for proper digestion, vitamin production, and acquisition of nutrients. Gut colonization by microbiota is crucial for development and function of the intestinal immune system (reviewed in Reference [2]). The colonization takes place at delivery while the newborn passes through the birth canal and during breast-feeding from the already colonized mother. In case the colonization occurs at a later age, the immune response to various stimuli is decreased and never recovers the levels observed in animals kept in standard conditions [3]. Moreover, commensal microbiota contribute to gut homeostasis by inducing region-dependent mucosal transcription [4]. The composition of microbial populations is influenced during a lifetime by the host’s diet, antibiotics usage, and genetic background of the host (reviewed in Reference [5]). Interestingly, a single gene polymorphism may impact the bacterial population diversity in the intestine [6].

Oral tolerance to various bacterial and food antigens develops in mice during the neonatal period and cannot be fully restored in adult originally germ-free (GF) animals upon bacterial colonization [7]. The absence of immune tolerance in GF animals resulted in allergies and inflammations or metabolic disorders such as diabetes (reviewed in Reference [8]). The link between the gut colonization and diverse disorders including autoimmune diseases has been observed in humans (reviewed in Reference [9]). Moreover, several recent studies documented microbiome changes during intestinal cancerogenesis or involvement of intestinal dysbiosis in cancer development [10,11,12]. Importantly, chronic inflammation associated with altered immune tolerance towards gut microbiota might result in disruption of the mucosal layer barrier function leading to inflammatory bowel disease (IBD) [13]. Additionally, chronic inflammation represents a risk factor in colitis-associated carcinoma of the colon and rectum [colorectal carcinoma (CRC)] [14]. Deciphering the impact of commensal microbiota on biological processes in the gut provides an important insight into the function of this organ in homeostatic or pathological conditions. 

The expression profiles of gut tissue obtained from conventionally reared (CR) or GF mice documented a profound effect of the microbiota on gene expression [3,4,15]. Interestingly, colonization of GF animals by the microbiota provokes dynamic alterations in gene expression prior to reaching homeostasis [4,16]. Nevertheless, only a limited number of studies—including the seminal article of Hooper and colleagues [17]—provided detailed characterization of gene expression profiling after monocolonization with a single bacterial strain [18]. 

In this study, we employed GF mice monocolonized either with the *Escherichia coli* (*E. coli*) O6K13 or Nissle 1917 strain to study mucosal gene expression upon exposure to a single bacterial type. The *E.coli* O6K13 strain belongs to antigen K13-producing uropathogenic bacteria that express ovalbumin to induce the local immune response [19]. The Nissle 1917 probiotic strain produces neither pathogenic adhesion factors, nor enterotoxins or cytotoxins. Consequently, the strain does not exhibit invasiveness or uropathogenic characteristics. Moreover, a therapeutic effect of *E. coli* Nissle 1917 bacteria in long-term remission of ulcerative colitis has been documented [20,21]. The molecular mechanisms responsible for the Nissle 1917 probiotic effects are mostly unclear, and the effect of both strains of bacteria on gene expression in the intestinal mucosa has not been studied in detail. Additionally, expression analysis was performed using total RNA isolated from colon organoids derived from CR, GF, and monoassociated mice. Development of three-dimensional cell cultures, so-called organoids, represents one of the most important technological advances in intestinal research over the past decade [22]. This technology allows generation of sphere-like structures from resected intestinal and colonic crypts or from single intestinal stem cell [23]. Organoids contain a stem cell population that self-renews and differentiates, generating all main cell types present in the intestinal epithelium. Moreover, the organoids resemble the intestinal architecture, and thus they faithfully reproduce some of the key features of the organ [24,25]. Additionally, since the organoids are formed from epithelial cells only, the analyzed RNA is not “contaminated” by RNA molecules obtained from other cell types. Thus, expression profiling of organoids can potentially bring new insights into the impact of microbiota on the epithelial cells. The obtained datasets were evaluated using the Enricher gene library online tool (http://amp.pharm.mssm.edu/Enrichr/). The analysis revealed which biological processes or cell types are affected by the absence or low complexity of commensal microbiota in the mouse intestine. 

## 2. Results and Discussion

### 2.1. Absence of Commensal Microbiota in the Intestine Mainly Affects the Mucosal Immune System and Metabolism

Expression profiling was performed using total RNA isolated from scraped mucosal lining of the middle and distal parts of the small intestine (further referred to as SI-middle, SI-distal, respectively) and from the colon. Of note, RNA obtained from the proximal small intestine was partially degraded, and was excluded from the analysis. The tissues were obtained from GF and CR mice and mice monoassociated with the *E. coli* O6K13 or Nissle 1917 strain, further referred to as ‘N’ and ‘O’ mice, respectively. RNA isolated from at least four littermates per each group was analyzed using cDNA microarrays. Initial evaluation was focused on genes whose expression changed significantly (expression intensity of the given gene changed more than twice between samples and Storey’s q-value < 0.05) in the comparison of CR and GF mice. The analysis revealed that 89, 195, and 94 genes were differentially expressed in the SI-middle, SI-distal, and colonic segments, respectively (Supplementary Table S1). We used the Enricher gene analysis tool to assign the genes to the ‘Gene Ontology Biological Process’ (GO BP) categories. Enricher provides an optimized algorithm for Gene Set Enrichment Analysis (GSEA) [26] with unique evaluation of statistical significance using deviation from the expected rank method [27]. As expected, in the small intestine, the majority of GO BPs were associated with the adaptive or innate immune response. Nevertheless, the ‘positive regulation of cell adhesion mediated by integrin’ GO BP was on the top of the GO BP list when genes changed in CR vs. GF mice were analyzed. Thus, we utilized the BioGPS gene annotation portal (http://biogps.org/) to investigate tissue and cell-specific expression of genes assigned to this GO BP [chemokine (C-C motif) ligand 5 (*Ccl5*); NCK Associated Protein 1 Like (*Nckap1l*), C-X-C Motif Chemokine Ligand 13 (*Cxcl13*)] in more detail. Interestingly, all these genes are highly expressed in various immune cells. Therefore, we extended the analysis and assigned the obtained gene sets to the ‘Cell Types/Mouse Gene Atlas’ (CT/MGA) category of the Enricher tool. This confirmed that indeed, in the intestinal mucosa, the majority of genes with the most significantly altered expression encode proteins produced in hematopoietic cells (predominantly follicular B cells) or tissue (Supplementary Figure S1). Moreover, since the expression level of these genes was mainly reduced in GF mice, this confirmed the previously published results that gut microbiota are indispensable for the establishment and maintenance of the mucosal immune system [28,29]. In contrast, only a limited number of genes were upregulated in GF mice. Gut microbiota carry out metabolic functions through hydrolysis and fermentation of dietary polysaccharides and fibers. Consequently, they increase energy extraction from the food [30]. Additionally, gut microbiota modulate the proliferation and differentiation of epithelial cells, mainly by producing short chain fatty acids (e.g., butyrate) [31]. In accordance with these metabolic and trophic functions, the absence of microbiota influenced expression of genes involved in the transport and metabolism of nutrients. Commensal microbiota, by producing reactive oxygen species (ROS), inactivate redox-sensitive dual specificity phosphatase (Dusp) enzymes in intestinal epithelial cells. Dusp inactivation leads to increased mitogen-activated protein kinase (MAPK) signaling in epithelial cells [32]. Therefore, the ‘inactivation of MAPK activity’ GO BP term in the GF intestine possibly refers to upregulation of the *Dusp1/6* genes (Supplementary Figure S1). In the colon, a significant decrease was noticed in the expression of genes encoding enzymes or enzymatic modulators expressed in several metabolic organs, including the pancreas, intestine, stomach, and liver. For example, reduced expression was noted for the pancreatic lipase related protein 1/2 (*Pnliprp1/2*) gene that encodes triglyceride lipase; colipase (*Clps*) encoding a cofactor of pancreatic lipase essential for efficient lipid hydrolysis, and phospholipase A2, group IIA (*Pla2g2a*) that encodes extracellular enzyme hydrolyzing phosphoglycerides [33]. Consequently, corresponding CT/MGA categories were top ranked in the Enricher tool results. This was in concordance with previous studies showing decreased adipocyte counts and lipogenesis accompanied by low levels of triglycerides in microbiota-free mice [34]. *Clps* and *Pnliprp2* represent components of the lipid absorption system; both genes were upregulated in the ileum of GF mice upon monocolonization with *Bacteroides thetaiotaomicron* [17]. Pla2g2a is secreted by goblet cells [35], i.e., a cell lineage dominantly present in the colon. El-Aidy and colleagues proposed that *Pla2g2a* expression might be a component of an innate defense response during bacterial colonization [4]. In addition, in agreement with previously published results, in CR mice we noted upregulation of other protective molecules, e.g., lysozyme 1/2 (*Lys1/2*) and resistin-like beta (*Retnlb*), in the small intestine and colon, respectively (Supplementary Table S1). However, unlike previously published results, in the comparison of CR vs. GF intestine, we did not observe any differences in cell proliferation or toll-like receptor (TLR) and type 1 interferon (IFN) signaling [3,4]. This discrepancy might be explained by the fact that the genes encoding proliferation markers or components of the TLR and IFN pathways were mainly upregulated during (or after) “conventionalization” of originally GF mice. In contrast, we performed expression profiling of mucosal samples obtained from mice that had been reared in conventional conditions for at least two generations. In conclusion, increased cell proliferation or TLR and IFN-mediated signaling might be a part of the temporary response during conventionalization.

Production of several mRNAs encoding signaling molecules were upregulated in the GF colon including bone morphogenic protein 3 (*Bmp3*) and hormones somatostatin (*Sst*) and glucagon (*Glg*). The increased levels of glucagon possibly reflect changes in the number or composition of colonic enteroendocrine cells [36,37]. Among the most upregulated genes in all regions of the GF intestine was angiopoietin-like 4 [(*Angptl4*); alternative name fasting-induced adipocyte factor (*Fiaf*)]. The gene encodes a secreted protein with pleiotropic functions in the organism [38]. Angptl4 blocks lipoprotein lipase-mediated storage of triglycerides in adipocytes and its expression in the small intestine is decreased upon GF mice recolonization [34,39,40,41]. Additionally, Angptl4 is required for functional partitioning of the postnatal intestinal lymphatic and blood vessels. Moreover, it promotes survival [42] or inhibits proliferation and migration of endothelial cells [43]. *ANGPTL4* expression is activated by peroxisome proliferator activated receptors (PPARs). Aronsson and co-workers showed that factor(s) secreted from probiotic *Lactobacillus paracasei* F19 (F19) bacteria induce ANGPTL4 production in human colon cancer cells or in gnotobiotic mice after monocolonization with F19 bacteria [44]. Interestingly, an increase in the Angptl4 mRNA and protein levels was observed in CR mice upon F19 supplementation to a high-fat diet. Nevertheless, we observed that the *Angptl4* expression levels are higher in GF when compared to CR mice, indicating alternative signaling mechanism(s) involved in *Angptl4* upregulation.

Very recently, Duszka and colleagues described an interesting phenomenon related to the response of intestinal cells to caloric restriction [45]. The response resulted in the polarization of small intestinal mucosa cell gene expression characterized by upregulation or downregulation of genes involved in the metabolic and immune/inflammatory pathways, respectively. Strikingly, Shulzhenko and colleagues described a similar phenomenon in B-cell-deficient mice. The authors showed that the B-cell-deficient (or IgA-deficient) intestinal epithelium triggers its own protective mechanisms by upregulating the IFN-inducible immune signaling pathway and simultaneously repressing (some) metabolic functions [46]. We attempted to track a similar response in tissue samples obtained from GF (and monoassociated) mice; however, this effort has not been successful. In fact, the overwhelming majority of transcripts underrepresented in the GF or gnotobiotic intestine were associated with gene expression typical of cells of the immune system. Duszka and colleagues analyzed the duodenum mucosa, and we performed expression profiling in more distal regions of the gastrointestinal tract. Thus, we cannot exclude the possibility that tissue regional differences might provoke different responses of epithelial cells upon caloric restriction. However, based on the experiments using CR and GF mice of various genetic backgrounds, Shulzhenko et al. concluded that microbiota are essential for epithelial cell reprograming in B-cell/IgA-deficient animals. In line with these facts, we prefer the hypothesis that the presence of microbiota is essential for epithelium reprograming upon caloric restriction or dysfunction of the immune system.

### 2.2. Monoassociation Does Not Restore the Mucosal Immune System

**Figure 1:**
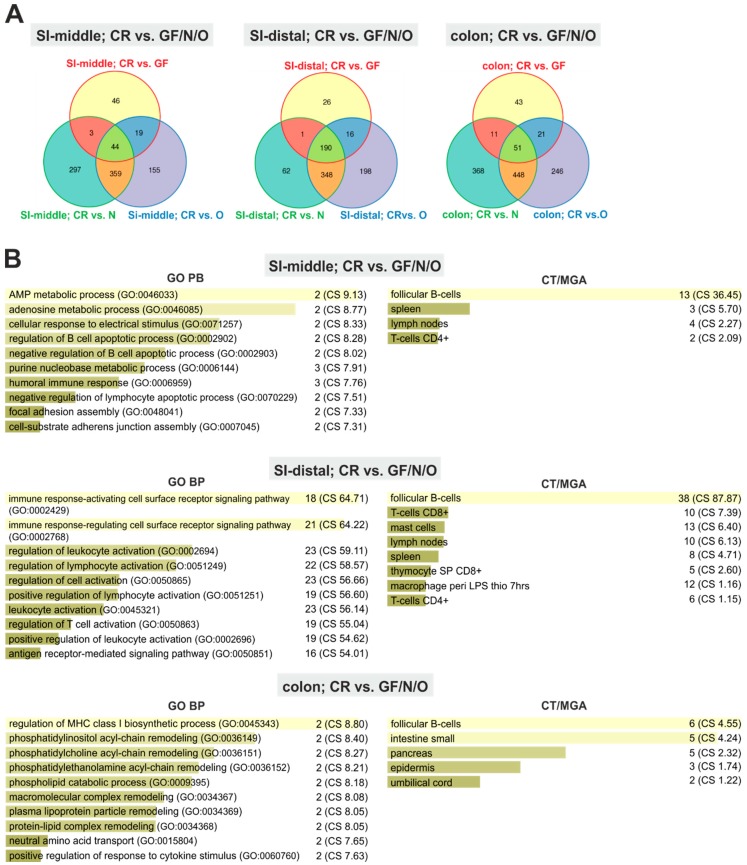
The immune system is not reconstituted upon monoassociation with a single *E. coli* strain. (**A**) Venn diagrams indicating numbers of gene probes differentially expressed in the middle (SI-middle) and distal (SI-distal) parts of the small intestine and in the colon of GF, N, and O animals when compared with CR mice (significance criterion: q < 0.05; |log FC| ≥ 1). The corresponding annotated genes are listed in Supplementary Table S2. (**B**) Genes expressed differentially in the analyzed groups of mice were analyzed using ‘Gene Ontology Biological Processes’ (GO BPs) and ‘Cell Types/Mouse Gene Atlas’ (CT/MGA) categories in Enricher datasets. The results were sorted according to the combined score (CS; CS is computed by multiplying the log of *p*-value obtained from the Fisher’s exact test by the z-score of the deviation from the expected rank). A maximum of ten GO BPs and CT/MGA categories containing at least two differentially expressed genes and with CS ≥ 1 is shown; the coloring of the columns corresponds to the significance of the individual columns in the graph. The number of genes in the particular category is indicated by the number before the parenthesis. |log FC|, absolute value of the binary logarithm of relative expression intensity.

Subsequently, we identified genes that distinguish the mucosa of GF, N, and O mice from the mucosa of CR animals. In the small intestinal segments, the majority of transcripts underrepresented in all three (i.e., GF, N, and O) tissues encoded proteins related to the immune response or proteins expressed in immune cells, indicating that colonization with a single *E.coli* strain is not sufficient to (fully) restore the mucosa-associated immune system (Figure 1 and Supplementary Table S2). The results supported the notion that only a restricted number of the segmented filamentous bacteria might recapitulate the immune-inducing effect of complex conventional microbiota [18].

Interestingly, only several genes were upregulated in the GF/N/O small intestine, including *Angptl4* (in SI-distal). In the colon, the genes with the most decreased expression were linked to the lipid metabolism (e.g., *Pnliprp2*, *Pla2g2a*, and *Pla2g4ac*) and reduced numbers of immune cells, as estimated from decreased expression of the *Cd74*, histocompatibility 2, class II antigen A, beta 1 (*H2-Ab1*), *H2-Eb1*, lymphocyte antigen 6 complex, locus E (*Ly6e*), and NLR family CARD domain containing 5 (*Nlrc5*) genes. Interestingly, the absence or low complexity of commensal microbiota resulted in decreased expression of angiogenin, ribonuclease A family, member 4 (*Ang4*) mRNA encoding a Paneth, or goblet cell-derived peptide with anti-microbial properties [47]. It was reported previously that *Ang4* expression was induced in the small intestine upon monoassociation with *Bacteroides thetaiotaomicron*, indicating (some) functional similarities of various bacterial species used for the gut colonization [17]. A very small number of genes (nine in total) were enriched in the GF/N/O colon, including *Sst* and *Bmp1*. Of note, only the zinc finger protein 326 (Z*fp326*) gene encoding a nuclear protein with unknown function was expressed inversely in the GF (upregulated in comparison to C mice) and N/O (downregulated) colon (Supplementary Table S2).

### 2.3. Genes Uniquely Expressed in the Intestine of GF Mice

**Figure 2:**
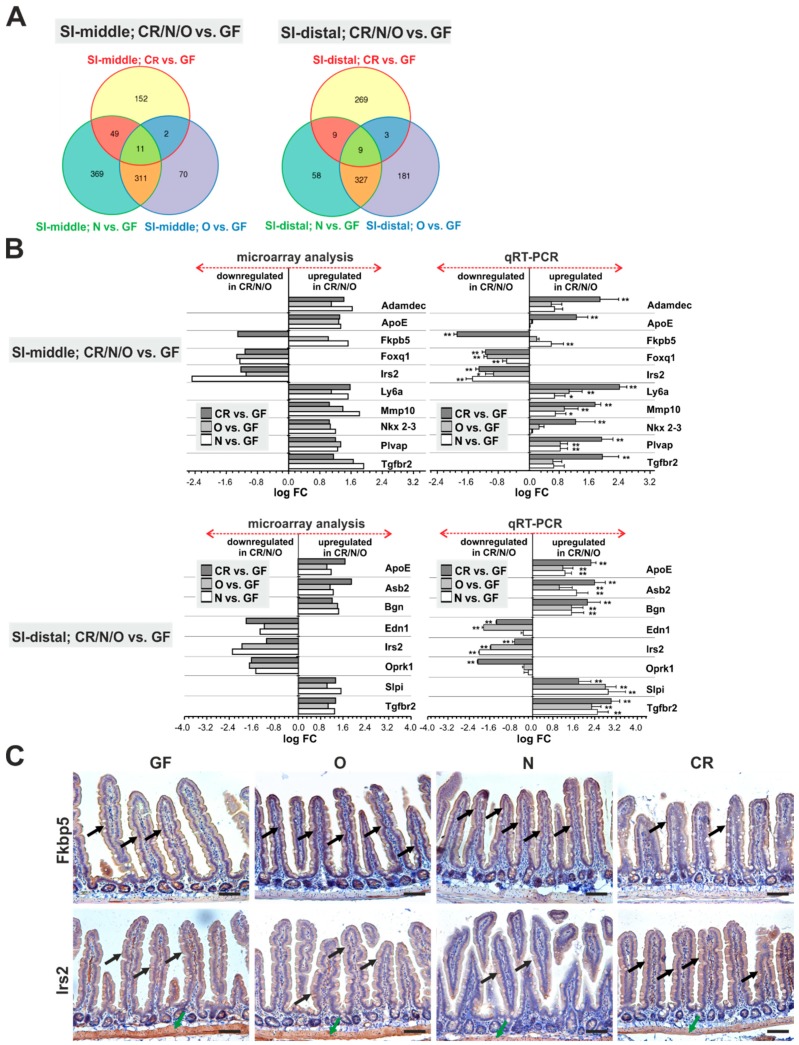
A limited number of genes are uniquely expressed in the GF small intestine. (**A**) Venn diagrams comparing gene expression profiles in small intestinal segments of CR, N, and O animals when contrasted with the gene expression profile obtained in GF mice. Eleven gene probes (representing 10 annotated genes) and 9 gene probes (representing 8 annotated genes) were differentially expressed in the SI-middle or SI-distal segment, respectively (selection criterion: |log FC| ≥ 1; *p* < 0.05 in at least two of three comparisons). The genes are listed in Supplementary Table S3. (**B**) Validation of cDNA microarray by qRT-PCR. Diagrams depict expression analysis of the indicated genes performed by cDNA microarray hybridization (left) or by qRT-PCR (right). Normalized fluorescence signal obtained in the respective gene probe upon hybridization with RNA isolated from GF mice was set to 1. Results of qRT-PCR were normalized to ubiquitin B (*Ubb*); the expression level of the respective gene in the mucosa of GF mice was set to 1. Four mice from each group were analyzed. Error bars represent SDs. Corresponding CT values are listed in Supplementary Table S4; * *p* < 0.05; ** *p* < 0.01 [one-way Analysis of Variance (ANOVA) test]. (**C**) Immunohistochemical detection of Fkbp5 and Irs2 using 3,3’-diaminobenzidine (DAB) staining (brownish precipitate) in the SI-middle part using specimens obtained from animals of the indicated experimental group. Both proteins are mainly produced in epithelial cells (black arrows). In addition, prominent Irs2 staining was also observed in the smooth muscle layer (green arrows). Representative images of specimens counterstained with hematoxylin are shown. Scale bar: 0.15 mm.

Next, we selected genes whose expression was changed significantly in GF mice when compared to animals harboring (any) gut microbiota, i.e., CR, N, and O mice. In the small intestinal mucosa, 11 and 9 gene probes were identified in the SI-middle and SI-distal segment, respectively, representing 12 different genes (Figure 2A, Supplementary Table S3). Subsequent qRT-PCR analysis confirmed the results of microarray hybridization with the exception of insulin receptor substrate 2 (*Irs2*) (Figure 2B). Since immunohistochemical (IHC) staining (Figure 2C) revealed that Irs2 is—besides epithelial cells—highly expressed in the intestinal muscle layer, the inconsistency of the results might be explained by contamination of (some) mucosal samples by muscle cells. In this particular contrast, the genes downregulated in the GF small intestine encoded secreted metalloproteinases ADAM-like, decysin 1 (*Adamdec1*) and matrix metallopeptidase 10 (*Mmp10/stromelysin-2*), apolipoprotein E (*ApoE*) essential for the catabolism of triglyceride-rich lipoproteins [48], and stem cell antigen-1/lymphocyte antigen 6 complex, locus A (*Sca-1/Ly6a*). *Sca-1* is expressed in multiple hematopoietic cells, where it is upregulated by interferon-alpha-mediated signaling [49]. Nevertheless, *Sca-1* gene expression in the intestinal epithelium at homeostatic conditions has not been reported. Therefore, *Sca-1* downregulation possibly indicated incomplete development of the mucosal immune system in the GF intestine. Additional genes downregulated in the GF small intestine were plasmalemma vesicle associated protein (*Plvap*) encoding an endothelial cell-specific protein [50], ankyrin repeat and SOCS box-containing 2 (*Asb2*) encoding E3 ubiquitin ligase highly expressed in immature dendritic cells [51], biglycan (*Bgn*) producing pericellular matrix proteoglycan produced in macrophages [52], and NK2 transcription factor related, locus 3 (*Nkx2-3*). Interestingly, the homeodomain-containing transcription factor Nkx2-3 has been linked to IBD [53,54]. Additionally, GF tissue produced less transforming growth factor, beta receptor II (*Tgfbr2*) known for its involvement in epithelial cells differentiation [55] and secretory leukocyte peptidase inhibitor (*Slpi*). Slpi functions as a secreted inhibitor of serine proteases protecting the intestinal epithelium [56]. In addition, Slpi possesses antibiotic activity and its expression is induced by microbial products [57] or is increased in the inflamed mucosa in ulcerative colitis patients [58]. Only the FK506 binding protein 5 (*Fkbp5*) gene encoding anti-inflammatory immunophilin with peptidyprolyl isomerase activity [59,60] was expressed inversely in the CR (downregulated) and N/O (upregulated in comparison to GF mice) small intestine (Figure 2B,C; Supplementary Table S3). Interestingly, in experimental piglets, *Fkbp5* expressional changes are dependent on the composition of gut microbiota [61]. Finally, three genes were upregulated in the GF small intestine, forkhead box Q1 (*Foxq1*) encoding transcription factor FOXQ1 that regulates epithelial-mesenchymal transition (EMT) in various carcinomas [62,63], and additionally, opioid receptor kappa 1 (*Oprk1*) and endothelin 1 (*Edn1*). Strikingly, Yu and colleagues observed *EDN1* gene repression by NKX2-3 in human intestinal endothelial cells [64]. Furthermore, inverse expression (i.e., *NKX2-3* upregulation and *EDN1* downregulation) was found in IBD patients [64,65].

**Figure 3:**
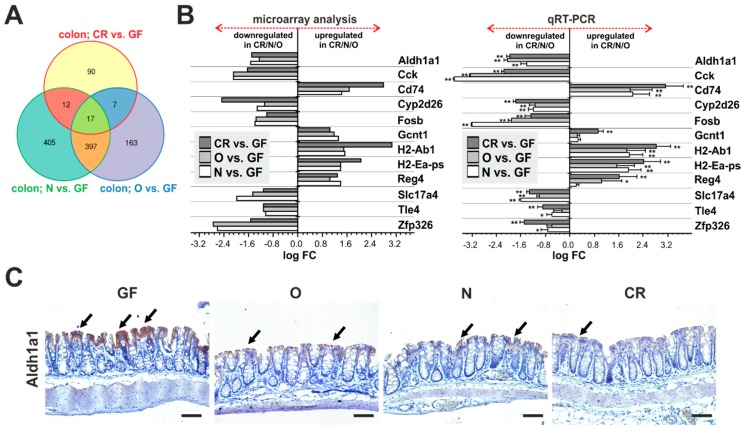
Aldh1a1 represents a robust marker of the GF colon. (**A**) A Venn diagram comparing gene expression profiles of the colonic mucosa of CR, N, and O animals when contrasted with the gene expression profile obtained in GF mice. According to the selection criterion (|log FC| ≥ 1; *p* < 0.05), 17 gene probes representing 12 annotated genes were identified. The genes are listed in Supplementary Table S3. (**B**) Validation of cDNA microarray by qRT-PCR. Comparison of expression analysis of the indicated genes performed by cDNA microarray hybridization (left) or by qRT-PCR (right). Samples obtained from four mice from each group were analyzed, as described in Figure 2B. Corresponding CT values are listed in Supplementary Table S4; * *p* < 0.05; ** *p* < 0.01 (one-way ANOVA test). (**C**) Immunohistochemical detection showing increased production of Aldh1a1 in differentiated epithelial cells (black arrows) of the colon of GF mice. Representative images of specimens counterstained with hematoxylin are shown. Scale bar: 0.15 mm.

In the colonic mucosa, 17 gene probes were identified representing 12 differentially expressed transcripts as validated by qRT-PCR (Figure 3A,B, Supplementary Table S3). Of five genes with decreased expression in GF mice, four were (functionally) linked to immune cells [*Cd74*, glucosaminyl (N-acetyl) transferase 1, core (*Gcnt1*), *2H2-Ab1*, and *H2-Ea-ps*]. The fifth gene, regenerating islet-derived family, member 4 (*Reg4*), encodes small secreted lectin that is expressed in the gastrointestinal tract especially in enteroendocrine cells [66,67]. REG4 functions as an anti-apoptotic tissue regeneration-promoting factor [68]. In the mouse intestine, *Reg4* expression (and expression of the related *Reg3β* and *Reg3γ* genes) was induced by gut-colonizing microbiota during postnatal development [15,69].

Genes significantly upregulated in GF animals included cytochrome P450 family member *Cyp2d26*, sodium/phosphate co-transporter solute carrier family 17, member 4 (*Slc17a4*), *Zfp326*, and aldehyde dehydrogenase family 1, subfamily A1 (*Aldh1a1*), that encodes the cytosolic enzyme involved in alcohol metabolism. It has been suggested that human ALDH1A1 represents one of the markers of so-called cancer stem cells [70,71,72]. However, in the mouse colon, Aldh1a1 was detected in differentiated epithelial cells at the surface of the tissue (and not in the crypts where intestinal stem cells reside). Aldh1a1 staining was remarkably increased in GF mice, confirming the result of mRNA expression profiling (Figure 3C). Additionally, we observed upregulation of the cholecystokinin (*Cck*), FBJ osteosarcoma oncogene B (*Fosb*) and transducin-like enhancer of split 4 (*Tle4*) genes. Transcription factor Fosb and co-repressor Tle4 are two nuclear proteins with no direct link to the gut physiology; *Cck* encodes a gut hormone functioning as the satiation signal in response to absorbed fatty acids upon luminal lipid hydrolysis [73]. In the mouse, cholecystokinin is predominantly secreted from enteroendocrine cells located in the proximal small intestine [74]. Thus, its expression in the colon is rather unexpected. However, in humans, cholecystokinin is produced in the colon, where it modulates the organ motility [75].

Finally, we compared the expression profiles of RNA isolated from the mucosa colonized with two different *E. coli* strains. Strikingly, three and no genes displayed significant expression changes in the SI-middle and SI-distal segment of the small intestine, respectively. The genes—showing reduced expression in N mice—were the sodium-dependent phosphate transporter solute carrier family 34, member 2 (*Slc34a2*), the putative heme transporter major facilitator superfamily domain containing 7C (*Mfsd7c*), and the steroid 5 alpha-reductase 1 (*Srd5a1*) enzyme catalyzing conversion of testosterone into dihydrotestosterone. In contrast, expression of 69 genes was upregulated in the colonic mucosa of N mice when compared to O mice. The majority of the genes were related to adipose tissue and lipid metabolism [e.g., adipogenin (*Adig*), adiponectin (*Adipoq*), cell death-inducing DFFA-like effector c (*Cidec*), *Pnliprp2*, perilipin 4 (*Plin4*), and leptin (*Lep*)] (Supplementary Table S5). As reported previously, monoassociation with the *E. coli* O6K13 strain promoted colonic inflammation in both the acute and chronic inflammation model [76]. It was suggested that probiotic bacteria of the Nissle 1917 strain protect the intestine indirectly by preventing its colonization by potential mucosal pathogens. Additionally, the strain possibly exerts immune-response-related function(s) by either suppressing inflammation or enhancing the epithelial defense and/or barrier functions [77,78]. However, the Enricher-based analysis of the differentially produced transcripts showed no signs of increased inflammation or immune cell infiltration in the O intestine when compared to N mice (Supplementary Figure S2). Thus, the observed differences in the colonic gene expression upon monoassociation might indicate (together with the effect of specific bacteria on mucosal transcription) the overall experimental animal’s fitness.

### 2.4. Expression Analysis of Organoids Derived from CR, GF, and Monoassociated Mice

**Figure 4:**
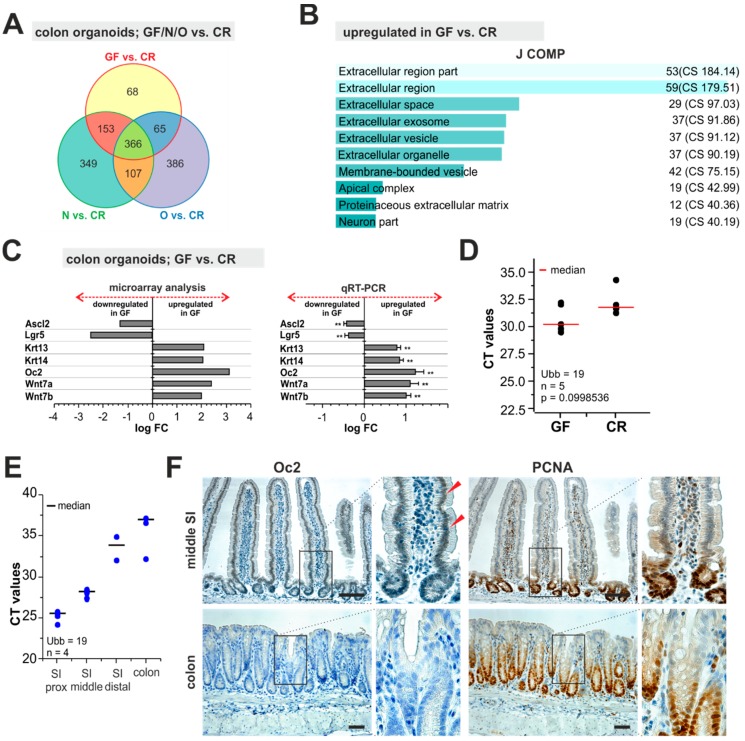
Oc2 expression was elevated in organoids derived from the gnotobiotic colon (**A**) A Venn diagram comparing gene expression profiles of colon organoids derived from GF, N, and O animals when compared to colon organoids derived from CR mice; 366 gene probes representing 285 annotated genes were identified. The genes are listed in Supplementary Table S6. (**B**) Enricher analysis of 138 genes upregulated in GF vs. CR colon organoids. The diagram shows top ten categories of the Jensen Compartment (J COMP; https://compartments.jensenlab.org/Search). (**C**) Validation of cDNA microarray by qRT-PCR. Diagrams depict expression analysis of the indicated genes in GF vs. CR colon organoids performed by cDNA microarray hybridization (left) or by qRT-PCR analysis (right). Normalized fluorescence signal obtained in the respective gene probe upon hybridization with RNA isolated from CR mice was set to 1. Results of the qRT-PCR analysis were normalized to *Ubb*; the expression level of the respective gene in the mucosa of CR mice was set to 1. Organoids derived from at four two mice were used; qRT-PCR reactions were run in technical triplicates. Error bars represent SDs. Corresponding CT values are listed in Supplementary Table S4; ** *p* < 0.01 (one-way ANOVA test). (**D**) Expression of the *Oc2* gene in the colon mucosa of CR and GF mice. Results were normalized to *Ubb* and the cycle threshold (CT) value of *Ubb* was arbitrarily set to 19. Five mice from each group were analyzed; median of CT values is indicated by a red line. The indicated *p* value was calculated using a one-way ANOVA test. (**E**) Oc2 expression levels along the rostro-caudal axis of the intestine. The diagram shows results of qRT-PCR using total RNA isolated from the mucosal scratches of the proximal (SI-prox), middle (SI-middle) and distal part (SI-distal) of the small intestine and from the middle portion of the colon of CR mice. Total RNA was obtained from 4 animals. Results were normalized to *Ubb* and the CT value of *Ubb* was arbitrarily set to 19; median of CT values is indicated by a grey line. (**F**) Immunohistochemical detection of Oc2 and proliferating cell nuclear antigen (PCNA) in the small intestine (jejunum) and middle part of the colon. The specimens were obtained from CR animals. Notice Oc2-positive cell nuclei in the small intestinal epithelial cells (red arrowheads). Specimens were counterstained with hematoxylin; boxed areas are magnified at the right. Scale bar: 0.15 mm.

Crypts obtained from the colon of CR, GF, and gnotobiotic mice were embedded in matrigel and cultured for seven days, then split and cultured for another five days. Total RNA isolated from the growing organoids was employed for expression profiling using DNA microarrays. Strikingly, in mutual comparison of organoids derived from monoassociated animals, none of genes passed the significance criteria (|logFC| ≥ 1; q < 0.05,), indicating a similar pattern of gene expression. In contrast, 366 gene probes representing 285 genes were differentially expressed in gnotobiotic when compared to CR mice (Figure 4A and Supplementary Table S6). This implied that despite the fact the that colonic epithelial cells were cultured for almost two weeks in vitro, they retained features related to their original in vivo settings. We further focused on the comparison of organoids derived from CR and GF mice. Interestingly, among 87 genes downregulated in GF mice we found genes encoding markers of intestinal stem cells (ISCs), leucine-rich repeat-containing G-protein-coupled receptor 5 (*Lgr5*) [79], and achaete-scute complex homolog 2 (*Ascl2*) [80]. *Lgr5* and *Ascl2* represent target genes of the Wnt/β-catenin pathway [80], which is essential for the self-renewing capacity of ISCs [81]. Thus, the finding implied that the Wnt pathway was impaired in the gnotobiotic organoids. Contrary to this assumption, *Wnt7a* and *Wnt7b* were among 138 genes upregulated in the GF colon organoids. However, the indicated Wnt ligands possibly trigger the so-called non-canonical, i.e., β-catenin-independent Wnt pathway [82,83,84], whose effects on ISCs have not been characterized. When we analyzed the differentially expressed genes using the Enricher tool, the most significantly changed GO BP terms were related to the extracellular space or cell interaction with the environment. The corresponding genes (upregulated in GF organoids) included secreted or membrane-associated proteins, extracellular matrix (ECM) components, ECM remodeling enzymes, or cytoskeletal proteins mediating cellular shape and cell interaction with ECM [e.g., *Bmp1*; collagen, type XVIII, alpha 1 (*Col18a1*); cytokeratins 4, 6B, 13, 14, and 84 (*Krt4/6B/13/14/84*); disintegrin-like and metallopeptidase (reprolysin type) with thrombospondin type 1 motif, and 15 (*Adamts15*); laminin subunit alpha 3 and gamma 2 (*Lama3*, *Lamc2*), kallikrein related-peptidase 15 (*Klk15*), and tenascin C (*Tnc*), etc.] (Figure 4B,C; Supplementary Table S6).

In comparison of differentially expressed genes either in the mucosa or organoids obtained from GF and CR mice, only six genes were common for both comparison; genes downregulated in the GF mucosa and organoids were: *Pnliprp2*, *Pla2g2a*, *Cd177*, and *Glipr2*; upregulated genes were: *Bmp1* and *Angptl4*. Whether this (relatively) low overlap between the two datasets was influenced by different complexity of the analyzed samples, i.e., mucosa versus epithelial organoids, or by changes elicited by the culture conditions remains to be determined.

One of the genes consistently upregulated in organoids derived from gnotobiotic colon was the One Cut Homeobox 2 (*Oc2*) gene encoding an evolutionarily conserved transcription factor (Figure 4C and Supplementary Table S6). Nevertheless, brief inspection of the microarray data did not indicate significant upregulation of the gene in intestinal tissue of GF or monocolonized mice. Subsequent qRT-PCR analysis displayed the increased *Oc2* expression levels in mucosal samples obtained from GF mice. Nevertheless, the statistical significance was not reached, probably due to the large variability of individual mucosal samples (Figure 4D). Oc2 is involved in development of neural tissues and retina [85,86,87]. Moreover, the factor participates in fetal intestinal development [88,89]. In the adult intestine, Oc2 is produced predominantly in the proximal portion of the small intestine [90,91]. Indeed, qRT-PCR analysis in mucosal scratches from CR mice showed decreasing *Oc2* mRNA levels along the rostro-caudal axis of the intestine (Figure 4E). Correspondingly, IHC staining showed Oct2-positive nuclei mainly in the epithelium lining of the proximal part of the small intestine. The protein was present in the majority of epithelial cells including crypt-resident ISCs, transit-amplifying (TA) cells, and in differentiated cells present on the villi. However, in the colon, Oc2 staining was detectable neither in CR nor in GF mice. Thus, although the qRT-PCR analysis showed increased Oc2 expression in the GF colon, the level of Oc2 protein was still below the detection limit of IHC staining (Figure 4F and data not shown).

## 3. Material and Methods

### 3.1. Experimental Animals, Ethics Statement

Housing of mice and in vivo experiments were performed in compliance with the European Communities Council Directive of 24 November 1986 (86/609/EEC) and national and institutional guidelines. Animal care and experimental procedures were approved by the Animal Care Committee of the Institute of Molecular Genetics (Ref. No. 180/2010). The expression profiling experiments were performed using adult mice (average age 13 weeks) of the BALB/c strain reared in GF or conventional conditions. Generation and housing of GF and monoassociated mice were described previously [76]. Mice of the second and subsequent generations after monoassociation were used in the study. Fecal samples were monthly collected and evaluated for the presence of bacteria. For both *E. coli*-monocolonized strains, bacterial counts were in the range of 10^9^–10^10^ colony forming units (CFU)/g wet feces weight during the mouse rearing period.

### 3.2. Tissue Isolation and Organoid Cultures

For mucosal scratches, at least six animals from each group of mice were euthanized by cervical dislocation, and the small intestines and colons were removed. The small intestine was divided into three pieces representing the proximal, middle, and distal parts; the colon was processed in one piece. The intestinal segments were opened longitudinally and washed in cold phosphate-buffered saline (PBS). The intestinal mucosa was scraped from the inner intestinal surface using a lancet. For organoid cultures, the colon crypts of four littermates from each group of animals were isolated and cultured as described previously [23,24]. Briefly, colon organoids were embedded in a Matrigel (growth factor reduced, phenol red free; BD Biosciences, San Jose, CA, USA) drop and cultured in basal organoid culture medium [advanced Dulbecco’s modified Eagle’s medium/F12 (adDMEM/F12)] supplemented with penicillin/streptomycin, 10 mmol/L HEPES, Glutamax, 1× N2, 1× B27 (all from Invitrogen, Carlsbad, CA, USA), 1 mmol/L N-acetylcysteine (Sigma-Aldrich, St. Louis, MO, USA). The medium also included mouse recombinant EGF (PeproTech, Rocky Hill, NJ, USA) and the conditioned media obtained from cells producing mouse R-spondin1 (dilution 1:10), mouse Noggin (1:10), and mouse Wnt3a ligand (1:1; Wnt3a-producing cells were kindly donated by M. Maurice, University Medical Center Utrecht, Utrecht, The Netherlands).

### 3.3. Microarray and qRT-PCR Analysis

Tissues or harvested organoids were immediately homogenized in RNA Blue (Top-Bio, Vestec, Czech Republic) and total RNA was isolated according to the manufacturer’s instructions. The total RNA was measured with a NanoDrop ND-1000 spectrophotometr (Thermo Fisher Scientific, Waltham, MA, USA) and the quality of isolated mRNA was checked using Agilent Bioanalyzer 2100 (Agilent, Santa Clara, CA, USA); RNAs with RNA integrity number (RIN) above 8 were further processed. For each sample, 250 ng of total mRNA was reverse transcribed and amplified using Illumina TotalPrep Amplification Kit (AMIL 1791, Thermo Fisher Scientific). A total of 750 ng of cDNA was analyzed by MouseRef-8 v2.0 Expression BeadChip (Illumina, San Diego, CA, USA). All steps were performed according to the respective manufacturer’s protocols. The analysis of mucosal scratches was performed in four biological replicates for GF and CR mice and five to six biological replicates for monocolonized mice. The analysis of organoids was performed in four biological replicates. Arrays were scanned using Illumina BeadArray Reader (a component of Illumina BeadStation500). The raw data were (pre)processed using GenomeStudio software (version 1.9.0.24624; Illumina) and further analyzed using the packages oligo [92] and limma [93] of Bioconductor [94] within the R environment [95]. Briefly, the transcription profiles were background corrected using a normal-exponential model, quantile normalized, and variance stabilized using base 2 logarithmic transformations. A moderated t-test was used to detect differentially expressed transcripts. Storey’s q-value [96] less than 0.05 and a minimally twofold change in expression intensity were required to consider genes as differentially transcribed. The MIAME compliant data was deposited to the ArrayExpress [97] database at EMBL-EBI (www.ebi.ac.uk/arrayexpress) under accession numbers E-MTAB-7726 (intestinal mucosa) and E-MTAB-7723 (intestinal organoids).

RNA samples from each group were further reverse transcribed by RevertAid Reverse Transcriptase (Thermo Fisher Scientific) and qRT-PCR was performed using the LightCycler 480 apparatus. SYBR Green I Master Mix (Roche Applied Science, Penzberg, Germany) was used for intestinal mucosa samples and LightCycler 480 Probes Master and Universal ProbeLibrary (UPL) hydrolysis probes were used for colon organoid samples. The expression levels were normalized to the ubiquitin B (*Ubb*) and glyceraldehyde 3-phosphate dehydrogenase (*Gapdh*) housekeeping genes, and the expression of another housekeeping gene, β-actin (*Actb*), was also included as a control. Primers and UPL probes used for qRT-PCR are listed in Supplementary Table S7. To evaluate the results of qRT-PCR analyses, we used a one-way Analysis of Variance (ANOVA) test, followed by the Dunnett’s Multiple Comparison test when comparing results obtained from individual groups of mice.

### 3.4. Gene Enrichment Analysis

The Enricher gene profiling web tool (http://amp.pharm.mssm.edu/Enrichr) was used to analyze differentially expressed genes.

### 3.5. Immunohistochemistry and Antibodies

Immunohistochemical staining was performed as described previously [98]. Briefly, tissues were fixed in 4% (*v*/*v*) formaldehyde in PBS and embedded in paraffin; antigen retrieval was performed in a steam bath by immersing the slides into 10 mM citrate buffer pH 6.0. Treatment in 0.2% H_2_O_2_ (Sigma-Aldrich; stock 30%) in methanol (Merck, Kenilworth, NJ, USA) for 25 min was used to block endogenous peroxidase activity. After 3 × PBS wash, specimens were blocked using 5% serum (goat or rabbit; Vector Laboratories; diluted in PBS) and 1% bovine serum albumin (BSA; diluted in PBS; Sigma-Aldrich) for 2 h. Primary antibodies: anti-Aldh1a1 (rabbit monoclonal, ab52492, Abcam, Cambridge, UK), anti-Fkbp5 (goat polyclonal, AF4094, R&D Systems, Minneapolis, MN, USA), anti-Irs2 (rabbit monoclonal, ab134101, Abcam), anti-Onecut2 (sheep polyclonal, AF6294, R&D Systems), anti-PCNA (rabbit polyclonal, ab18197, Abcam). The primary antibody was applied overnight at 4 °C; next day, specimens were washed in PBS and incubated for 1 h in PBS containing 1% BSA and biotin-conjugated secondary antibodies (dilution 1:750; Biotin-XX Goat anti-Rabbit IgG, B-2770, Thermo Fisher Scientific; Biotin-XX Goat anti-Mouse IgG, #2763, Thermo Fisher Scientific; Biotin-XX Rabbit anti-Sheep IgG, #31840, Invitrogen). The signal was enhanced using the Vectastain ABC kit (Vector Laboratories, Burlingame, CA, USA) and developed in DAB (Sigma-Aldrich; 30 mg dissolved in 90 mL 50 mM Tris, pH 7.5, supplemented with H_2_O_2_ to final concentration 0.3%). Sections were counterstained with hematoxylin (PENTA, Prague, Czech Republic).

## 4. Conclusions

In this study, we determined the gene expression profiles of the mucosa isolated from the small intestine and colon of GF mice and animals monoassociated with single *E. coli* strains. We showed that the complete absence of commensal microbiota in the intestine mainly affected the mucosal immune system, adipose tissue, and lipid metabolism. The expression signature of the small intestinal mucosa of mice monoassociated with two different *E. coli* strains was almost identical. However, in the colon monoassociated with probiotic *E. coli* strain Nissle, more than 60 genes were upregulated in comparison to the mice colonized with *E. coli* pathogenic strain O6K13. The majority of the genes were related to adipose tissue, lipid metabolism, or to adipocyte differentiation. In contrast, no signs of increased inflammation or immune cell infiltration in animals monoassociated with *E. coli* pathogenic strain O6K13 were observed. The molecular mechanisms responsible for the Nissle 1917 probiotic effects remain unclear. Nevertheless, the study confirmed that monocolonization with the *E. coli* bacterium does exert an immune-inducing effect typical of complex conventional microbiota or of (some) filamentous bacterial strains.

Additionally, we analyzed the gene expression profiles in colon organoids derived from CR, GF, N, and O mice. Gene expression in organoids prepared from O and N mice differed negligibly; in contrast, multiple genes were differentially expressed in organoids derived from GF and CR mice. The differentially expressed genes included markers of ISCs, secreted or membrane-associated proteins, ECM components and remodeling enzymes, and cytoskeletal proteins mediating cellular shape and cell interaction with its environment. In the comparison of differentially expressed genes including RNA samples isolated from the mucosa or organoids obtained from GF and CR mice, only six genes were common to both comparison. Whether such a (relatively) low count was influenced by cell culture conditions or by different cellular complexity of the analyzed samples remains to be determined. Our results show that one of the “signs” indicating the GF status is increased expression of the *Angptl4* gene encoding a secreted inhibitor of lipoprotein lipase. Finally, one of the genes whose expression was elevated both in GF organoids and the gut mucosa encoded the Oc2 transcription factor. The mechanism of Oc2 upregulation in the colon of GF and monoassociated mice and its possible role in the tissue require further analysis.

## Supplementary Materials

Supplementary materials can be found at https://www.mdpi.com/1422-0067/20/7/1581/s1.
