# Supplementary material for: Unique Gene Expression Signatures in the Intestinal Mucosa and Organoids Derived from Germ-Free and Monoassociated Mice
Source: Int J Mol Sci. 2019 Mar 29;20(7):1581. doi: 10.3390/ijms20071581 (PMC6480644; doi:10.3390/ijms20071581)
Supplement: Supplementary file 1 [file ijms-20-01581-s001.pdf]

Supplementary Material

Supplementary Figure Legends

Supplementary Figure S1. Expression profiles of the intestinal mucosa obtained from CR and GF animals. Top two diagrams displayed in yellow shading show analysis of differentially expressed genes (significance criterion: q-value < 0.05; |log FC| ≥ 1) in all analyzed segments of the intestine using the GO BP and CT/MGA Enricher datasets. Bottom diagram in blue shading indicates analysis of genes upregulated in the GF intestine (significance criterion: q-value < 0.05; log FC|≥ 1) using the GO BP dataset. Individual genes are listed in Supplementary Table S1. Additional information is given in Fig. 1 legend.

Supplementary Figure S2. Analysis of differentially expressed genes in the colon of monoassociated mice. The most different GO BPs and CT/MGA categories in expression profiles of the colonic mucosa obtained from monoassociated mice. The significance criterion (q-value < 0.05; |log FC| ≥ 1) was passed by 72 gene probes (representing 69 annotated genes) that were analyzed as described in the Fig. 1 legend. The genes are listed in Supplementary Table S5.

Supplementary Table Legends

Supplementary Table S1. Comparison of expression profiles of the mucosa obtained from CR and GF mice

Supplementary Table S2. List of genes differentially expressed in GF, N, and O animals when compared to CR mice

Supplementary Table S3. List of genes differentially expressed in CR, N, and O animals when compared to GF mice

Supplementary Table S4. List of CT values obtained by qRT-PCR analysis

Supplementary Table S5. Comparison of monoassociated mice

Supplementary Table S6. List of genes differentially expressed in colon organoids derived from GF, N, and O mice when compared to CR mice

Supplementary Table S7. List of primers and UPL probes used for qRT-PCR

Supplementary Figure S1

all differentially expressed genes in CR vs. GF

GO BP

positive regulation of B cell proliferation (GO:0030890)	5 (CS 45.69)
positive regulation of lymphocyte proliferation (GO:0050671)	8 (CS 29.24)
regulation of MHC class I biosynthetic process (GO:0045343)	2 (CS 28.26)
regulation of B cell proliferation (GO:0030888)	5 (CS 24.61)
positive regulation of cell adhesion mediated by integrin (GO:0033630)	3 (CS 21.55)
regulation of T cell proliferation (GO:0042129)	5 (CS 20.99)
T cell migration (GO:0072678)	3 (CS 20.93)
positive regulation of T cell chemotaxis (GO:0010820)	2 (CS 19.13)
lymphocyte chemotaxis (GO:0048247)	5 (CS 18.83)
regulation of natural killer cell chemotaxis (GO:2000501)	2 (CS 18.53)

CT/MGA

follicular B-cells	26 (CS 72.24)
lymph nodes	9 (CS 9.27)
T-cells CD8+	6 (CS 6.56)
spleen	4 (CS 4.83)
thymocyte SP CD8+	4 (CS 4.47)
T-cells CD4+	4 (CS 4.04)
macrophage peri LPS thio 7hrs	8 (CS 3.35)
thymocyte DP CD4+CD8+	4 (CS 3.31)
mast cells	6 (CS 3.04)
microglia	2 (CS 2.39)

upregulated in GF vs. CR

GO BP

amino acid transmembrane transport (GO:0003333)	3 (CS 22.08)
neutral amino acid transport (GO:0015804)	3 (CS 20.94)
amino acid transport (GO:0006865)	4 (CS 19.55)
carboxylic acid transmembrane transport (GO:1905039)	3 (CS 18.53)
nitrogen compound transport (GO:0071705)	4 (CS 17.67)
inactivation of MAPK activity (GO:0000188)	2 (CS 15.69)
negative regulation of lipase activity (GO:0060192)	2 (CS 15.36)
regulation of lipoprotein lipase activity (GO:0051004)	2 (CS 14.34)
peptidyl-tyrosine dephosphorylation (GO:0035335)	2 (CS 13.92)
glyceraldehyde-3-phosphate metabolic process (GO:0019682)	2 (CS 13.66)

Supplementary Figure S2

GO BP

brown fat cell differentiation (GO:0050873)	4 (CS 13.34)
fat cell differentiation (GO:0045444)	6 (CS 11.90)
muscle contraction (GO:0006936)	7 (CS 11.47)
muscle system process (GO:0003012)	7 (CS 9.49)
muscle filament sliding (GO:0030049)	3 (CS 5.49)
actin-myosin filament sliding (GO:0033275)	3 (CS 5.48)
cellular response to vitamin (GO:0071295)	2 (CS 5.47)
bone mineralization (GO:0030282)	3 (CS 5.41)
regulation of system process (GO:0044057)	7 (CS 5.26)
white fat cell differentiation (GO:0050872)	2 (CS 5.04)

CT/MGA

adipose brown	15 (CS 29.69)
adipose white	10 (CS 12.03)
hypothalamus	6 (CS 10.52)
mammary gland non-lactating	7 (CS 9.36)
skeletal muscle	10 (CS 6.03)
dorsal root ganglia	7 (CS 4.05)
cerebral cortex prefrontal	5 (CS 2.88)
pancreas	4 (CS 2.66)
cerebellum	4 (CS 2.20)

TF ChIP-seq

PPARG (PMID 19300518) 3T3-L1 mouse cell line	9 (CS 51.434)
IKZF1 (PMID 21737484) HCT116 human cell line	3 (CS 13.76)
HTT (PMID 18923047) striatum-derived human cell lines	6 (CS 12.53)
CEBPB (PMID 23403033) mouse liver cells	5 (CS 12.34)
NR1I2 (PMID 20693526) mouse liver cells	8 (CS 11.30)
NR0B1 (PMID 18358816) mouse embryonic stem cells	13 (CS 10.89)
TP53 (PMID 18474530) U2OS human cell line	8 (CS 10.67)
REST (PMID 18959480) mouse embryonic stem cells	17 (CS 10.40)
CDX2 (PMID 19796622) mouse embryonic stem cells	4 (CS 10.29)
MEF2A (PMID 21415370) HL-1 mouse cell line	8 (CS 10.14)

Supplementary Table S1

Differentialy expressed genes (|logFC|≥1; q < 0.05) in the mucosa of the middle and distal part of the small intestine and colon of CR vs. GF mice (contrast: CR vs. GF)

ENTREZ No.	Symbol	Gene name	Log FC
14961	H2-Ab1	histocompatibility 2, class II antigen A, beta 1	3.69
14999	H2-DMb1	histocompatibility 2, class II, locus Mb1	3.50
16149	Cd74	CD74 antigen (invariant polypeptide of major histocompatibility complex, class II antigen-associated)	3.32
14969	H2-Eb1	histocompatibility 2, class II antigen E beta	2.96
14968	H2-Ea-ps	histocompatibility 2, class II antigen E alpha, pseudogene	2.47
12265	Ciita	class II transactivator	2.12
12511	Cd6	CD6 antigen	1.90
239743	Klhl6	kelch-like 6 (Drosophila)	1.89
15985	Cd79b	CD79B antigen	1.88
93695	Gpnmb	glycoprotein (transmembrane) nmb	1.86
69169	Faim3	Fas apoptotic inhibitory molecule 3	1.84
55985	Cxcl13	chemokine (C-X-C motif) ligand 13	1.69
17329	Cxcl9	chemokine (C-X-C motif) ligand 9	1.67
21838	Thy1	thymus cell antigen 1, theta	1.67
24108	Ubd	ubiquitin D	1.67
20304	Ccl5	chemokine (C-C motif) ligand 5	1.64
110454	Ly6a	lymphocyte antigen 6 complex, locus A	1.64
23833	Cd52	CD52 antigen	1.62
18947	Pnliprp2	pancreatic lipase-related protein 2	1.62
18985	Pou2af1	POU domain, class 2, associating factor 1	1.60
14204	Il4i1	interleukin 4 induced 1	1.59
16792	Laptm5	lysosomal-associated protein transmembrane 5	1.57
12526	Cd8b1	CD8 antigen, beta chain 1	1.55
17110	Lyz1	lysozyme 1	1.54
68891	Cd177	CD177 antigen	1.52
20303	Ccl4	chemokine (C-C motif) ligand 4	1.52
232889	Pla2g4c	phospholipase A2, group IVC (cytosolic, calcium-independent)	1.52
17105	Lyz2	lysozyme 2	1.49
11857	Arhgdib	Rho, GDP dissociation inhibitor (GDI) beta	1.49
11872	Art2b	ADP-ribosyltransferase 2b	1.48
12501	Cd3e	CD3 antigen, epsilon polypeptide	1.48
109791	Clps	colipase, pancreatic	1.47
17067	Ly6c1	lymphocyte antigen 6 complex, locus C1	1.47
12721	Coro1a	coronin, actin binding protein 1A	1.46
74096	Hvcn1	hydrogen voltage-gated channel 1	1.39
231932	Gimap7	GTPase, IMAP family member 7	1.38
15953	Ifi47	interferon gamma inducible protein 47	1.35
11472	Actn2	actinin alpha 2	1.34
67133	Gp2	glycoprotein 2 (zymogen granule membrane)	1.33
107321	Lpxn	leupaxin	1.33
18780	Pla2g2a	phospholipase A2, group IIA (platelets, synovial fluid)	1.33
20715	Serpina3g	serine (or cysteine) peptidase inhibitor, clade A, member 3G	1.31
14468	Gbp1	guanylate binding protein 1	1.30
17069	Ly6e	lymphocyte antigen 6 complex, locus E	1.29
20568	Slpi	secretory leukocyte peptidase inhibitor	1.28
12500	Cd3d	CD3 antigen, delta polypeptide	1.27
98752	Fcrla	Fc receptor-like A	1.26
74015	Fcho1	FCH domain only 1	1.26
16421	Itgb7	integrin beta 7	1.26

14469	Gbp2	guanylate binding protein 2	1.25
22379	Fmnl3	formin-like 3	1.24
17304	Mfge8	milk fat globule-EGF factor 8 protein	1.24
16913	Psmb8	proteasome (prosome, macropain) subunit, beta type 8 (large multifunctional peptidase 7)	1.23
231931	Gimap6	GTPase, IMAP family member 6	1.22
27261	Dok3	docking protein 3	1.21
16985	Lsp1	lymphocyte specific 1	1.21
27371	Sh2d2a	SH2 domain protein 2A	1.21
18301	Fxyd5	FXYD domain-containing ion transport regulator 5	1.20
16429	Itln1	intelectin 1 (galactofuranose binding)	1.20
16994	Ltb	lymphotoxin B	1.20
13041	Ctsw	cathepsin W	1.19
22364	Vpreb3	pre-B lymphocyte gene 3	1.19
22637	Zap70	zeta-chain (TCR) associated protein kinase	1.19
65256	Asb2	ankyrin repeat and SOCS box-containing 2	1.18
110168	Gpr18	G protein-coupled receptor 18	1.18
434341	Nlrc5	NLR family, CARD domain containing 5	1.18
12502	Cd3g	CD3 antigen, gamma polypeptide	1.16
16145	Igtp	interferon gamma induced GTPase	1.15
105855	Nckap1l	NCK associated protein 1 like	1.14
16498	Kcnab2	potassium voltage-gated channel, shaker-related subfamily, beta member 2	1.14
20621	Snn	stannin	1.12
17972	Ncf4	neutrophil cytosolic factor 4	1.11
53376	Usp2	ubiquitin specific peptidase 2	1.11
16407	Itgae	integrin alpha E, epithelial-associated	1.09
17896	Myl4	myosin, light polypeptide 4	1.09
72310	Nkg7	natural killer cell group 7 sequence	1.08
320484	Rasal3	RAS protein activator like 3	1.08
12260	C1qb	complement component 1, q subcomponent, beta polypeptide	1.06
13170	Dbp	D site albumin promoter binding protein	1.05
321019	Gpr183	G protein-coupled receptor 183	1.05
19419	Rasgrp1	RAS guanyl releasing protein 1	1.05
20491	Sla	src-like adaptor	1.05
72049	Tnfrsf13c	tumor necrosis factor receptor superfamily, member 13c	1.05
17084	Ly86	lymphocyte antigen 86	1.04
11816	Apoe	apolipoprotein E	1.03
12262	C1qc	complement component 1, q subcomponent, C chain	1.03
56743	Lat2	linker for activation of T cells family, member 2	1.03
226594	Rcsd1	RCSD domain containing 1	1.03
74191	P2ry13	purinergic receptor P2Y, G-protein coupled 13	1.02
215653	Rassf2	Ras association (RalGDS/AF-6) domain family member 2	1.02
24099	Tnfsf13b	tumor necrosis factor (ligand) superfamily, member 13b	1.00
384783	Irs2	insulin receptor substrate 2	-1.02
14377	G6pc	glucose-6-phosphatase, catalytic	-1.11
13120	Cyp4b1	cytochrome P450, family 4, subfamily b, polypeptide 1	-1.35
57875	Angptl4	angiopoietin-like 4	-2.25

Supplementary Table S1

Differentialy expressed genes (|logFC|≥1; q < 0.05) in the mucosa of the middle part of the small intestine of conventionally reared (CR) vs. germ-free (GF) mice (contrast: CR vs. GF)

ENTREZ No.	Symbol	Gene name	Log FC
93695	Gpnmb	glycoprotein (transmembrane) nmb	3.10
14961	H2-Ab1	histocompatibility 2, class II antigen A, beta 1	2.99
20568	Slpi	secretory leukocyte peptidase inhibitor	2.86
69169	Faim3	Fas apoptotic inhibitory molecule 3	2.82
14999	H2-DMb1	histocompatibility 2, class II, locus Mb1	2.74
16149	Cd74	CD74 antigen (invariant polypeptide of major histocompatibility complex, class II antigen-associated)	2.70
55985	Cxcl13	chemokine (C-X-C motif) ligand 13	2.46
14969	H2-Eb1	histocompatibility 2, class II antigen E beta	2.40
15985	Cd79b	CD79B antigen	2.23
11472	Actn2	actinin alpha 2	2.12
17067	Ly6c1	lymphocyte antigen 6 complex, locus C1	2.00
21838	Thy1	thymus cell antigen 1, theta	1.97
74096	Hvcn1	hydrogen voltage-gated channel 1	1.90
12511	Cd6	CD6 antigen	1.87
20440	St6gal1	beta galactoside alpha 2,6 sialyltransferase 1	1.81
14968	H2-Ea-ps	histocompatibility 2, class II antigen E alpha, pseudogene	1.80
17110	Lyz1	lysozyme 1	1.79
12265	Ciita	class II transactivator	1.76
22364	Vpreb3	pre-B lymphocyte gene 3	1.73
12721	Coro1a	coronin, actin binding protein 1A	1.61
21807	Tsc22d1	TSC22 domain family, member 1	1.61
20303	Ccl4	chemokine (C-C motif) ligand 4	1.58
12526	Cd8b1	CD8 antigen, beta chain 1	1.57
24108	Ubd	ubiquitin D	1.57
22379	Fmnl3	formin-like 3	1.52

17896	Myl4	myosin, light polypeptide 4	1.40
217169	Tns4	tensin 4	1.40
11629	Aif1	allograft inflammatory factor 1	1.37
74191	P2ry13	purinergic receptor P2Y, G-protein coupled 13	1.36
20304	Ccl5	chemokine (C-C motif) ligand 5	1.31
67876	Coq10b	coenzyme Q10 homolog B (S. cerevisiae)	1.30
231932	Gimap7	GTPase, IMAP family member 7	1.30
69772	Bdh2	3-hydroxybutyrate dehydrogenase, type 2	1.29
215653	Rassf2	Ras association (RalGDS/AF-6) domain family member 2	1.29
107321	Lpxn	leupaxin	1.28
381524	AI427809	expressed sequence AI427809	1.27
11486	Ada	adenosine deaminase	1.26
23959	Nt5e	5' nucleotidase, ecto	1.26
11565	Adssl1	adenylosuccinate synthetase like 1	1.24
103149	Upb1	ureidopropionase, beta	1.24
16421	Itgb7	integrin beta 7	1.23
16909	Lmo2	LIM domain only 2	1.23
12259	C1qa	complement component 1, q subcomponent, alpha polypeptide	1.20
12448	Ccne2	cyclin E2	1.19
102595	Plekho2	pleckstrin homology domain containing, family O member 2	1.19
320484	Rasal3	RAS protein activator like 3	1.19
12458	Ccr6	chemokine (C-C motif) receptor 6	1.17
110168	Gpr18	G protein-coupled receptor 18	1.17
108956	Apol7c	apolipoprotein L 7c	1.16
13036	Ctsh	cathepsin H	1.16
11872	Art2b	ADP-ribosyltransferase 2b	1.14
268567	Tmem229b	transmembrane protein 229B	1.14
74015	Fcho1	FCH domain only 1	1.13
56743	Lat2	linker for activation of T cells family, member 2	1.13
70785	Dennd1c	DENN/MADD domain containing 1C	1.12
233571	P2ry6	pyrimidinergic receptor P2Y, G-protein coupled, 6	1.11
20345	Selplg	selectin, platelet (p-selectin) ligand	1.11
13057	Cyba	cytochrome b-245, alpha polypeptide	1.10

19245	Ptp4a3	protein tyrosine phosphatase 4a3	1.10
13041	Ctsw	cathepsin W	1.07
72310	Nkg7	natural killer cell group 7 sequence	1.06
74131	Sash3	SAM and SH3 domain containing 3	1.05
22637	Zap70	zeta-chain (TCR) associated protein kinase	1.05
12766	Cxcr3	chemokine (C-X-C motif) receptor 3	1.03
15163	Hcls1	hematopoietic cell specific Lyn substrate 1	1.03
102545	Cmtm7	CKLF-like MARVEL transmembrane domain containing 7	1.01
78416	Rnase6	ribonuclease, RNase A family, 6	1.01
76527	Il34	interleukin 34	1.00
67603	Dusp6	dual specificity phosphatase 6	-1.02
12660	Chka	choline kinase alpha	-1.03
67603	Dusp6	dual specificity phosphatase 6	-1.03
26897	Acot1	acyl-CoA thioesterase 1	-1.04
66270	Fam134b	family with sequence similarity 134, member B	-1.04
84112	Sucnr1	succinate receptor 1	-1.04
212398	Frat2	frequently rearranged in advanced T-cell lymphomas 2	-1.06
224023	Klhl22	kelch-like 22 (Drosophila)	-1.09
234788	Slc38a8	solute carrier family 38, member 8	-1.10
13131	Dab1	disabled homolog 1 (Drosophila)	-1.18
12125	Bcl2l11	BCL2-like 11 (apoptosis facilitator)	-1.19
17195	Mbl2	mannose-binding lectin (protein C) 2	-1.22
74155	Errfi1	ERBB receptor feedback inhibitor 1	-1.23
50490	Nox4	NADPH oxidase 4	-1.23
14377	G6pc	glucose-6-phosphatase, catalytic	-1.35
17748	Mt1	metallothionein 1	-1.35
17750	Mt2	metallothionein 2	-1.35
217721	Mfsd7c	major facilitator superfamily domain containing 7C	-1.42
11865	Arntl	aryl hydrocarbon receptor nuclear translocator-like	-1.46
57875	Angptl4	angiopoietin-like 4	-1.90
20531	Slc34a2	solute carrier family 34 (sodium phosphate), member 2	-1.98

Supplementary Table S1

Differentialy expressed genes (|logFC|≥1; q < 0.05) in the mucosa of the distal part of the small intestine of CR vs. GF mice (contrast: CR vs. GF)

ENTREZ No.	Symbol	Gene name	Log FC
14961	H2-Ab1	histocompatibility 2, class II antigen A, beta 1	5.03
14999	H2-DMb1	histocompatibility 2, class II, locus Mb1	4.69
16149	Cd74	CD74 antigen (invariant polypeptide of major histocompatibility complex, class II antigen-associated)	4.35
14969	H2-Eb1	histocompatibility 2, class II antigen E beta	4.11
14968	H2-Ea-ps	histocompatibility 2, class II antigen E alpha, pseudogene	3.55
20304	Ccl5	chemokine (C-C motif) ligand 5	3.45
12501	Cd3e	CD3 antigen, epsilon polypeptide	3.06
24108	Ubd	ubiquitin D	3.01
12511	Cd6	CD6 antigen	2.98
12502	Cd3g	CD3 antigen, gamma polypeptide	2.84
14170	Fgf15	fibroblast growth factor 15	2.77
12265	Ciita	class II transactivator	2.66
16792	Laptm5	lysosomal-associated protein transmembrane 5	2.63
12500	Cd3d	CD3 antigen, delta polypeptide	2.53
12721	Coro1a	coronin, actin binding protein 1A	2.49
239743	Klhl6	kelch-like 6 (Drosophila)	2.49
12526	Cd8b1	CD8 antigen, beta chain 1	2.48
17105	Lyz2	lysozyme 2	2.46
23833	Cd52	CD52 antigen	2.41
17329	Cxcl9	chemokine (C-X-C motif) ligand 9	2.38
110168	Gpr18	G protein-coupled receptor 18	2.34
16407	Itgae	integrin alpha E, epithelial-associated	2.34
11857	Arhgdib	Rho, GDP dissociation inhibitor (GDI) beta	2.34
17110	Lyz1	lysozyme 1	2.32
13041	Ctsw	cathepsin W	2.31
21838	Thy1	thymus cell antigen 1, theta	2.27
231932	Gimap7	GTPase, IMAP family member 7	2.21
18985	Pou2af1	POU domain, class 2, associating factor 1	2.20
15985	Cd79b	CD79B antigen	2.19
16913	Psmb8	proteasome (prosome, macropain) subunit, beta type 8 (large multifunctional peptidase 7)	2.16
72310	Nkg7	natural killer cell group 7 sequence	2.15
18301	Fxyd5	FXYD domain-containing ion transport regulator 5	2.14
16985	Lsp1	lymphocyte specific 1	2.12
14468	Gbp1	guanylate binding protein 1	2.11
16421	Itgb7	integrin beta 7	2.06
55985	Cxcl13	chemokine (C-X-C motif) ligand 13	2.04
20303	Ccl4	chemokine (C-C motif) ligand 4	2.02
11872	Art2b	ADP-ribosyltransferase 2b	2.01
107321	Lpxn	leupaxin	2.00
19419	Rasgrp1	RAS guanyl releasing protein 1	2.00
105855	Nckap1l	NCK associated protein 1 like	1.98
27371	Sh2d2a	SH2 domain protein 2A	1.98
14469	Gbp2	guanylate binding protein 2	1.97
68891	Cd177	CD177 antigen	1.96
17304	Mfge8	milk fat globule-EGF factor 8 protein	1.96
16145	Igtp	interferon gamma induced GTPase	1.94
16994	Ltb	lymphotoxin B	1.94
22637	Zap70	zeta-chain (TCR) associated protein kinase	1.94

20230	Satb1	special AT-rich sequence binding protein 1	1.92
17304	Mfge8	milk fat globule-EGF factor 8 protein	1.90
65256	Asb2	ankyrin repeat and SOCS box-containing 2	1.89
27261	Dok3	docking protein 3	1.89
14204	Il4i1	interleukin 4 induced 1	1.89
231931	Gimap6	GTPase, IMAP family member 6	1.85
15953	Ifi47	interferon gamma inducible protein 47	1.85
20715	Serpina3g	serine (or cysteine) peptidase inhibitor, clade A, member 3G	1.85
16818	Lck	lymphocyte protein tyrosine kinase	1.82
320484	Rasal3	RAS protein activator like 3	1.78
11472	Actn2	actinin alpha 2	1.77
546546	Serpina3h	serine (or cysteine) peptidase inhibitor, clade A, member 3H	1.77
20491	Sla	src-like adaptor	1.77
21940	Cd27	CD27 antigen	1.75
16909	Lmo2	LIM domain only 2	1.75
16498	Kcnab2	potassium voltage-gated channel, shaker-related subfamily, beta member 2	1.75
93695	Gpnmb	glycoprotein (transmembrane) nmb	1.69
246177	Myo1g	myosin IG	1.69
17084	Ly86	lymphocyte antigen 86	1.68
16170	Il16	interleukin 16	1.67
11816	Apoe	apolipoprotein E	1.66
13537	Dusp2	dual specificity phosphatase 2	1.66
18826	Lcp1	lymphocyte cytosolic protein 1	1.65
12143	Blk	B lymphoid kinase	1.64
17972	Ncf4	neutrophil cytosolic factor 4	1.64
22271	Upp1	uridine phosphorylase 1	1.64
53376	Usp2	ubiquitin specific peptidase 2	1.60
20345	Selplg	selectin, platelet (p-selectin) ligand	1.58
54215	Cd160	CD160 antigen	1.57
12262	C1qc	complement component 1, q subcomponent, C chain	1.56
23871	Ets1	E26 avian leukemia oncogene 1, 5' domain	1.56
434341	Nlrc5	NLR family, CARD domain containing 5	1.56
12493	Cd37	CD37 antigen	1.55
17067	Ly6c1	lymphocyte antigen 6 complex, locus C1	1.55
22329	Vcam1	vascular cell adhesion molecule 1	1.55
30948	Bin1	bridging integrator 1	1.54
15000	H2-DMb2	histocompatibility 2, class II, locus Mb2	1.53
17698	Msn	moesin	1.53
18751	Prkcb	protein kinase C, beta	1.53
22379	Fmnl3	formin-like 3	1.52
58194	Sh3kbp1	SH3-domain kinase binding protein 1	1.52
12515	Cd69	CD69 antigen	1.50
19354	Rac2	RAS-related C3 botulinum substrate 2	1.50
74015	Fcho1	FCH domain only 1	1.48
229323	Gpr171	G protein-coupled receptor 171	1.46
18718	Pip4k2a	phosphatidylinositol-5-phosphate 4-kinase, type II, alpha	1.46
215653	Rassf2	Ras association (RalGDS/AF-6) domain family member 2	1.45
16428	Itk	IL2-inducible T-cell kinase	1.44
54354	Rassf5	Ras association (RalGDS/AF-6) domain family member 5	1.44
14007	Celf2	CUGBP, Elav-like family member 2	1.43
70785	Dennd1c	DENN/MADD domain containing 1C	1.43
226594	Rcsd1	RCSD domain containing 1	1.42
66995	Zcchc18	zinc finger, CCHC domain containing 18	1.42
22376	Was	Wiskott-Aldrich syndrome homolog (human)	1.41
15930	Ido1	indoleamine 2,3-dioxygenase 1	1.40
16643	Klrd1	killer cell lectin-like receptor, subfamily D, member 1	1.40
12522	Cd83	CD83 antigen	1.38
13713	Elk3	ELK3, member of ETS oncogene family	1.38

16797	Lat	linker for activation of T cells	1.37
12145	Cxcr5	chemokine (C-X-C motif) receptor 5	1.36
15163	Hcls1	hematopoietic cell specific Lyn substrate 1	1.35
67742	Samsn1	SAM domain, SH3 domain and nuclear localization signals, 1	1.35
21813	Tgfbr2	transforming growth factor, beta receptor II	1.34
16598	Klf2	Kruppel-like factor 2 (lung)	1.33
17069	Ly6e	lymphocyte antigen 6 complex, locus E	1.33
16541	Napsa	napsin A aspartic peptidase	1.33
17896	Myl4	myosin, light polypeptide 4	1.32
74131	Sash3	SAM and SH3 domain containing 3	1.32
243374	Gimap8	GTPase, IMAP family member 8	1.30
232201	Arhgap25	Rho GTPase activating protein 25	1.30
18636	Cfp	complement factor properdin	1.29
13170	Dbp	D site albumin promoter binding protein	1.29
14281	Fos	FBJ osteosarcoma oncogene	1.29
70719	Hmha1	histocompatibility (minor) HA-1	1.29
15001	H2-Oa	histocompatibility 2, O region alpha locus	1.29
13449	Dok2	docking protein 2	1.28
321019	Gpr183	G protein-coupled receptor 183	1.28
15277	Hk2	hexokinase 2	1.28
24099	Tnfsf13b	tumor necrosis factor (ligand) superfamily, member 13b	1.28
13057	Cyba	cytochrome b-245, alpha polypeptide	1.27
12458	Ccr6	chemokine (C-C motif) receptor 6	1.26
224109	Lrrc33	leucine rich repeat containing 33	1.26
14960	H2-Aa	histocompatibility 2, class II antigen A, alpha	1.25
56743	Lat2	linker for activation of T cells family, member 2	1.24
226652	Arhgap30	Rho GTPase activating protein 30	1.24
22324	Vav1	vav 1 oncogene	1.24
13421	Dnase1l3	deoxyribonuclease 1-like 3	1.22
259277	Klk8	kallikrein related-peptidase 8	1.22
12508	Cd53	CD53 antigen	1.21
21354	Tap1	transporter 1, ATP-binding cassette, sub-family B (MDR/TAP)	1.21
238377	Gpr68	G protein-coupled receptor 68	1.19
68682	Slc44a2	solute carrier family 44, member 2	1.19
12483	Cd22	CD22 antigen	1.18
14130	Fcgr2b	Fc receptor, IgG, low affinity IIb	1.18
67905	Ppm1m	protein phosphatase 1M	1.17
17085	Ly9	lymphocyte antigen 9	1.16
19265	Ptprcap	protein tyrosine phosphatase, receptor type, C polypeptide-associated protein	1.16
98752	Fcrla	Fc receptor-like A	1.14
100182	Akna	AT-hook transcription factor	1.13
15894	Icam1	intercellular adhesion molecule 1	1.13
19245	Ptp4a3	protein tyrosine phosphatase 4a3	1.13
78416	Rnase6	ribonuclease, RNase A family, 6	1.12
67102	D16Ertd472e	DNA segment, Chr 16, ERATO Doi 472, expressed	1.11
15900	Irf8	interferon regulatory factor 8	1.11
239827	Pigz	phosphatidylinositol glycan anchor biosynthesis, class Z	1.11
76438	Rftn1	raftlin lipid raft linker 1	1.11
216445	Arhgap9	Rho GTPase activating protein 9	1.11
102545	Cmtm7	CKLF-like MARVEL transmembrane domain containing 7	1.10
74191	P2ry13	purinergic receptor P2Y, G-protein coupled 13	1.10
12263	C2	complement component 2 (within H-2S)	1.09
14357	Dtx1	deltex 1 homolog (Drosophila)	1.09
17259	Mef2b	myocyte enhancer factor 2B	1.09
227659	Slc2a6	solute carrier family 2 (facilitated glucose transporter), member 6	1.09
12524	Cd86	CD86 antigen	1.08
241732	Tspyl3	TSPY-like 3	1.08

21939	Cd40	CD40 antigen	1.07
22778	Ikzf1	IKAROS family zinc finger 1	1.07
18707	Pik3cd	phosphatidylinositol 3-kinase catalytic delta polypeptide	1.07
73341	Arhgef6	Rac/Cdc42 guanine nucleotide exchange factor (GEF) 6	1.07
232984	B3gnt8	UDP-GlcNAc:betaGal beta-1,3-N-acetylglucosaminyltransferase 8	1.07
268857	Nlrc3	NLR family, CARD domain containing 3	1.06
108101	Fermt3	fermitin family homolog 3 (Drosophila)	1.04
16176	Il1b	interleukin 1 beta	1.04
243910	Nfkbid	nuclear factor of kappa light polypeptide gene enhancer in B-cells inhibitor, delta	1.04
12443	Ccnd1	cyclin D1	1.03
16197	Il7r	interleukin 7 receptor	1.02
14667	Gm2a	GM2 ganglioside activator protein	1.00
16154	Il10ra	interleukin 10 receptor, alpha	1.00
16453	Jak3	Janus kinase 3	1.00
18126	Nos2	nitric oxide synthase 2, inducible	1.00
68344	Tmem174	transmembrane protein 174	-1.01
12408	Cbr1	carbonyl reductase 1	-1.02
218454	Lhfpl2	lipoma HMGIC fusion partner-like 2	-1.04
18143	Npas2	neuronal PAS domain protein 2	-1.05
71733	Susd2	sushi domain containing 2	-1.05
11828	Aqp3	aquaporin 3	-1.10
50934	Slc7a8	solute carrier family 7 (cationic amino acid transporter, y+ system), member 8	-1.14
70113	Odf3b	outer dense fiber of sperm tails 3B	-1.15
71904	Paqr7	progestin and adipoQ receptor family member VII	-1.17
234788	Slc38a8	solute carrier family 38, member 8	-1.41
18387	Oprk1	opioid receptor, kappa 1	-1.64
64454	Slc5a4b	solute carrier family 5 (neutral amino acid transporters, system A), member 4b	-1.77
13614	Edn1	endothelin 1	-1.83
71898	Apol9b	apolipoprotein L 9b	-1.85
14377	G6pc	glucose-6-phosphatase, catalytic	-1.98
27273	Pdk4	pyruvate dehydrogenase kinase, isoenzyme 4	-2.04
57875	Angptl4	angiopoietin-like 4	-3.06

Supplementary Table S1

Differentialy expressed genes (|logFC|≥1; q < 0.05) in the mucosa of the large intestine of CR vs. GF mice

(contrast: CR vs. GF)

ENTREZ No.	Symbol	Gene name	Log FC
18947	Pnliprp2	pancreatic lipase-related protein 2	5.16
18946	Pnliprp1	pancreatic lipase related protein 1	4.46
109791	Clps	colipase, pancreatic	3.73
232889	Pla2g4c	phospholipase A2, group IVC (cytosolic, calcium-independent)	3.61
16429	Itln1	intelectin 1 (galactofuranose binding)	3.29
18780	Pla2g2a	phospholipase A2, group IIA (platelets, synovial fluid)	3.20
14999	H2-DMb1	histocompatibility 2, class II, locus Mb1	3.08
14961	H2-Ab1	histocompatibility 2, class II antigen A, beta 1	3.07
16149	Cd74	CD74 antigen (invariant polypeptide of major histocompatibility complex, class II antigen-associated)	2.90
14969	H2-Eb1	histocompatibility 2, class II antigen E beta	2.37
219033	Ang4	angiogenin, ribonuclease A family, member 4	2.29
57263	Retnlb	resistin like beta	2.28
68468	Ly6g6c	lymphocyte antigen 6 complex, locus G6C	2.25
15430	Hoxd10	homeobox D10	2.12
14968	H2-Ea-ps	histocompatibility 2, class II antigen E alpha, pseudogene	2.06
12265	Ciita	class II transactivator	1.93
69065	Chac1	ChaC, cation transport regulator-like 1 (E. coli)	1.88
110454	Ly6a	lymphocyte antigen 6 complex, locus A	1.82
17341	Bhlha15	basic helix-loop-helix family, member a15	1.80
15953	Ifi47	interferon gamma inducible protein 47	1.80
19367	Rad9	RAD9 homolog (S. pombe)	1.73
17069	Ly6e	lymphocyte antigen 6 complex, locus E	1.71
67198	Spats2l	spermatogenesis associated, serine-rich 2-like	1.68
68891	Cd177	CD177 antigen	1.66
66120	Fkbp11	FK506 binding protein 11	1.59
23797	Akt3	thymoma viral proto-oncogene 3	1.59
26366	Ceacam10	carcinoembryonic antigen-related cell adhesion molecule 10	1.58
12332	Capg	capping protein (actin filament), gelsolin-like	1.53
23882	Gadd45g	growth arrest and DNA-damage-inducible 45 gamma	1.52
233038	Nccrp1	non-specific cytotoxic cell receptor protein 1 homolog (zebrafish)	1.52
434341	Nlrc5	NLR family, CARD domain containing 5	1.48
13105	Cyp2d9	cytochrome P450, family 2, subfamily d, polypeptide 9	1.47
234577	Cpne2	copine II	1.46
384009	Glipr2	GLI pathogenesis-related 2	1.40
229933	Clca5	chloride channel calcium activated 5	1.37
216188	Aldh1l2	aldehyde dehydrogenase 1 family, member L2	1.32
67709	Reg4	regenerating islet-derived family, member 4	1.31
11872	Art2b	ADP-ribosyltransferase 2b	1.29
80859	Nfkbiz	nuclear factor of kappa light polypeptide gene enhancer in B-cells inhibitor, zeta	1.29
209588	Sectm1a	secreted and transmembrane 1A	1.24
12124	Bik	BCL2-interacting killer	1.23
16913	Psmb8	proteasome (prosome, macropain) subunit, beta type 8 (large multifunctional peptidase 7)	1.22
103149	Upb1	ureidopropionase, beta	1.22
67475	Ero1lb	ERO1-like beta (S. cerevisiae)	1.21
104601	Mycbpap	MYCBP associated protein	1.20
21818	Tgm3	transglutaminase 3, E polypeptide	1.20
74015	Fcho1	FCH domain only 1	1.18
27356	Insl6	insulin-like 6	1.14
20731	Spink4	serine peptidase inhibitor, Kazal type 4	1.12

12409	Cbr2	carbonyl reductase 2	1.11
12263	C2	complement component 2 (within H-2S)	1.11
216456	Gls2	glutaminase 2 (liver, mitochondrial)	1.10
70536	Qpct	glutaminyl-peptide cyclotransferase (glutaminyl cyclase)	1.09
317652	Klk15	kallikrein related-peptidase 15	1.09
56045	Samhd1	SAM domain and HD domain, 1	1.07
100041194	Ahnak2	AHNAK nucleoprotein 2	1.05
53376	Usp2	ubiquitin specific peptidase 2	1.05
76267	Fads1	fatty acid desaturase 1	1.04
14537	Gcnt1	glucosaminyl (N-acetyl) transferase 1, core 2	1.04
16402	Itga5	integrin alpha 5 (fibronectin receptor alpha)	1.04
16622	Klk1b5	kallikrein 1-related peptidase b5	1.02
56773	Chst5	carbohydrate (N-acetylglucosamine 6-O) sulfotransferase 5	1.00
15432	Hoxd12	homeobox D12	1.00
214897	Csnk1g1	casein kinase 1, gamma 1	-1.00
18789	Papola	poly (A) polymerase alpha	-1.00
76722	Ckmt2	creatine kinase, mitochondrial 2	-1.02
14282	Fosb	FBJ osteosarcoma oncogene B	-1.02
22051	Trip6	thyroid hormone receptor interactor 6	-1.06
16939	Lor	loricrin	-1.09
21888	Tle4	transducin-like enhancer of split 4, homolog of Drosophila E(spl)	-1.10
14526	Gcg	glucagon	-1.12
56365	Clcnkb	chloride channel Kb	-1.13
319848	Slc17a4	solute carrier family 17 (sodium phosphate), member 4	-1.13
230163	Aldob	aldolase B, fructose-bisphosphate	-1.17
19252	Dusp1	dual specificity phosphatase 1	-1.17
11812	Apoc1	apolipoprotein C-I	-1.19
258458	Olfr165	olfactory receptor 165	-1.22
22239	Ugt8a	UDP galactosyltransferase 8A	-1.33
54367	Zfp326	zinc finger protein 326	-1.40
13829	Epb4.9	erythrocyte protein band 4.9	-1.47
11668	Aldh1a1	aldehyde dehydrogenase family 1, subfamily A1	-1.49
12153	Bmp1	bone morphogenetic protein 1	-1.50
68404	Nrn1	neuritin 1	-1.57
235674	Acaa1b	acetyl-Coenzyme A acyltransferase 1B	-1.58
66184	Rps4y2	ribosomal protein S4, Y-linked 2	-1.59
17829	Muc1	mucin 1, transmembrane	-1.62
12424	Cck	cholecystokinin	-1.64
57875	Angptl4	angiopoietin-like 4	-1.79
110075	Bmp3	bone morphogenetic protein 3	-1.88
74338	Slc6a19	solute carrier family 6 (neurotransmitter transporter), member 19	-1.88
20604	Sst	somatostatin	-1.94
381204	Naaladl1	N-acetylated alpha-linked acidic dipeptidase-like 1	-2.05
30962	Slc7a9	solute carrier family 7 (cationic amino acid transporter, y+ system), member 9	-2.20
76279	Cyp2d26	cytochrome P450, family 2, subfamily d, polypeptide 26	-2.49

Supplementary Table S2

Differentialy expressed genes (|logFC|≥1; q < 0.05) in the colonic mucosa of CR vs. GF and monoassociated (N or O) mice (contrast: CR vs.GF/N/O)

ENTREZ No.	Symbol	Gene name	Change
216188	Aldh1l2	aldehyde dehydrogenase 1 family, member L2	up
219033	Ang4	angiogenin, ribonuclease A family, member 4	up
11812	Apoc1	apolipoprotein C-I	down
11872	Art2b	ADP-ribosyltransferase 2b	up
17341	Bhlha15	basic helix-loop-helix family, member a15	up
12153	Bmp1	bone morphogenetic protein 1	down
12332	Capg	capping protein (actin filament), gelsolin-like	up
12409	Cbr2	carbonyl reductase 2	up
68891	Cd177	CD177 antigen	up
16149	Cd74	gen (invariant polypeptide of major histocompatibility complex, class II antigen-	up
69065	Chac1	ChaC, cation transport regulator-like 1 (E. coli)	up
12265	Ciita	class II transactivator	up
229933	Clca5	chloride channel calcium activated 5	up
76279	Cyp2d26	cytochrome P450, family 2, subfamily d, polypeptide 26	down
13829	Epb4.9	erythrocyte protein band 4.9	down
66120	Fkbp11	FK506 binding protein 11	up
23882	Gadd45g	growth arrest and DNA-damage-inducible 45 gamma	up
216456	Gls2	glutaminase 2 (liver, mitochondrial)	up
14961	H2-Ab1	histocompatibility 2, class II antigen A, beta 1	up
14999	H2-DMb1	histocompatibility 2, class II, locus Mb1	up
14969	H2-Eb1	histocompatibility 2, class II antigen E beta	up
15953	Ifi47	interferon gamma inducible protein 47	up
16429	Itln1	intelectin 1 (galactofuranose binding)	up
16939	Lor	loricrin	down
17069	Ly6e	lymphocyte antigen 6 complex, locus E	up
68468	Ly6g6c	lymphocyte antigen 6 complex, locus G6C	up
434341	Nlrc5	NLR family, CARD domain containing 5	up
18780	Pla2g2a	phospholipase A2, group IIA (platelets, synovial fluid)	up
232889	Pla2g4c	phospholipase A2, group IVC (cytosolic, calcium-independent)	up
18947	Pnliprp2	pancreatic lipase-related protein 2	up
70536	Qpct	glutaminyl-peptide cyclotransferase (glutaminyl cyclase)	up
19367	Rad9	RAD9 homolog (S. pombe)	up
66184	Rps4y2	ribosomal protein S4, Y-linked 2	down
74338	Slc6a19	solute carrier family 6 (neurotransmitter transporter), member 19	down
30962	Slc7a9	solute carrier family 7 (cationic amino acid transporter, y+ system), member 9	down
20604	Sst	somatostatin	down
54367	Zfp326	zinc finger protein 326	C_G: down<br>C_N/O: up

Supplementary Table S2

Differentialy expressed genes (|logFC|≥1; q < 0.05) in the mucosa of the middle part of the small intestine of conventionally reared (CR) vs. germ-free (GF) and monoassociated (N or O) mice (contrast: CR vs.GF/N/O)

ENTREZ No.	Symbol	Gene name	Change
11472	Actn2	actinin alpha 2	up
11486	Ada	adenosine deaminase	up
11565	Adssl1	adenylosuccinate synthetase like 1	up
12125	Bcl2l11	BCL2-like 11 (apoptosis facilitator)	down
12448	Ccne2	cyclin E2	up
12458	Ccr6	chemokine (C-C motif) receptor 6	up
12511	Cd6	CD6 antigen	up
16149	Cd74	CD74 antigen (invariant polypeptide of major histocompatibility complex, class II antigen-associated)	up
15985	Cd79b	CD79B antigen	up
12265	Ciita	class II transactivator	up
55985	Cxcl13	chemokine (C-X-C motif) ligand 13	up
69169	Faim3	Fas apoptotic inhibitory molecule 3	up
74015	Fcho1	FCH domain only 1	up
231932	Gimap7	GTPase, IMAP family member 7	up
93695	Gpnmb	glycoprotein (transmembrane) nmb	up
14961	H2-Ab1	histocompatibility 2, class II antigen A, beta 1	up
14999	H2-DMb1	histocompatibility 2, class II, locus Mb1	up
14969	H2-Eb1	histocompatibility 2, class II antigen E beta	up
16421	Itgb7	integrin beta 7	up
17195	Mbl2	mannose-binding lectin (protein C) 2	down
17750	Mt2	metallothionein 2	down
17896	Myl4	myosin, light polypeptide 4	up
23959	Nt5e	5' nucleotidase, ecto	up
215653	Rassf2	Ras association (RalGDS/AF-6) domain family member 2	up
20568	Slpi	secretory leukocyte peptidase inhibitor	up

20440	St6gal1	beta galactoside alpha 2,6 sialyltransferase 1	up
21838	Thy1	thymus cell antigen 1, theta	up
21807	Tsc22d1	TSC22 domain family, member 1	up
24108	Ubd	ubiquitin D	up
22364	Vpreb3	pre-B lymphocyte gene 3	up

Supplementary Table S2

Differentialy expressed genes (|logFC|≥1; q < 0.05) in the mucosa of the distal part of the small intestine of CR vs. GF and monoassociated (N or O) mice (contrast: CR vs.GF/N/O)

ENTREZ No.	Symbol	Gene name	Change
11472	Actn2	actinin alpha 2	up
57875	Angptl4	angiopoietin-like 4	down
71898	Apol9b	apolipoprotein L 9b	down
11828	Aqp3	aquaporin 3	down
232201	Arhgap25	Rho GTPase activating protein 25	up
226652	Arhgap30	Rho GTPase activating protein 30	up
216445	Arhgap9	Rho GTPase activating protein 9	up
11857	Arhgdib	Rho, GDP dissociation inhibitor (GDI) beta	up
11872	Art2b	ADP-ribosyltransferase 2b	up
232984	B3gnt8	UDP-GlcNAc:betaGal beta-1,3-N-acetylglucosaminyltransferase 8	up
30948	Bin1	bridging integrator 1	up
12143	Blk	B lymphoid kinase	up
12408	Cbr1	carbonyl reductase 1	down
20303	Ccl4	chemokine (C-C motif) ligand 4	up
20304	Ccl5	chemokine (C-C motif) ligand 5	up
12458	Ccr6	chemokine (C-C motif) receptor 6	up
54215	Cd160	CD160 antigen	up
68891	Cd177	CD177 antigen	up
21940	Cd27	CD27 antigen	up
12493	Cd37	CD37 antigen	up
12500	Cd3d	CD3 antigen, delta polypeptide	up
12501	Cd3e	CD3 antigen, epsilon polypeptide	up
12502	Cd3g	CD3 antigen, gamma polypeptide	up
21939	Cd40	CD40 antigen	up
23833	Cd52	CD52 antigen	up
12508	Cd53	CD53 antigen	up
12511	Cd6	CD6 antigen	up
12515	Cd69	CD69 antigen	up
16149	Cd74	CD74 antigen (invariant polypeptide of major histocompatibility complex, class II antigen-associated)	up
15985	Cd79b	CD79B antigen	up
12524	Cd86	CD86 antigen	up
12526	Cd8b1	CD8 antigen, beta chain 1	up
18636	Cfp	complement factor properdin	up
12265	Ciita	class II transactivator	up
102545	Cmtm7	CKLF-like MARVEL transmembrane domain containing 7	up
12721	Coro1a	coronin, actin binding protein 1A	up
13041	Ctsw	cathepsin W	up
55985	Cxcl13	chemokine (C-X-C motif) ligand 13	up
17329	Cxcl9	chemokine (C-X-C motif) ligand 9	up
12145	Cxcr5	chemokine (C-X-C motif) receptor 5	up
13057	Cyba	cytochrome b-245, alpha polypeptide	up
67102	D16Ertd472e	DNA segment, Chr 16, ERATO Doi 472, expressed	up
70785	Dennd1c	DENN/MADD domain containing 1C	up
13449	Dok2	docking protein 2	up
27261	Dok3	docking protein 3	up
13537	Dusp2	dual specificity phosphatase 2	up
13713	Elk3	ELK3, member of ETS oncogene family	up
23871	Ets1	E26 avian leukemia oncogene 1, 5' domain	up
14130	Fcgr2b	Fc receptor, IgG, low affinity IIb	up
74015	Fcho1	FCH domain only 1	up

98752	Fcrla	Fc receptor-like A	up
108101	Fermt3	fermitin family homolog 3 (Drosophila)	up
14170	Fgf15	fibroblast growth factor 15	up
22379	Fmnl3	formin-like 3	up
18301	Fxyd5	FXYD domain-containing ion transport regulator 5	up
14377	G6pc	glucose-6-phosphatase, catalytic	down
14468	Gbp1	guanylate binding protein 1	up
14469	Gbp2	guanylate binding protein 2	up
231931	Gimap6	GTPase, IMAP family member 6	up
231932	Gimap7	GTPase, IMAP family member 7	up
243374	Gimap8	GTPase, IMAP family member 8	up
93695	Gpnmb	glycoprotein (transmembrane) nmb	up
229323	Gpr171	G protein-coupled receptor 171	up
110168	Gpr18	G protein-coupled receptor 18	up
321019	Gpr183	G protein-coupled receptor 183	up
238377	Gpr68	G protein-coupled receptor 68	up
14961	H2-Ab1	histocompatibility 2, class II antigen A, beta 1	up
14999	H2-DMb1	histocompatibility 2, class II, locus Mb1	up
15000	H2-DMb2	histocompatibility 2, class II, locus Mb2	up
14968	H2-Ea-ps	histocompatibility 2, class II antigen E alpha, pseudogene	up
14969	H2-Eb1	histocompatibility 2, class II antigen E beta	up
15001	H2-Oa	histocompatibility 2, O region alpha locus	up
15163	Hcls1	hematopoietic cell specific Lyn substrate 1	up
15277	Hk2	hexokinase 2	up
70719	Hmha1	histocompatibility (minor) HA-1	up
15930	Ido1	indoleamine 2,3-dioxygenase 1	up
15953	Ifi47	interferon gamma inducible protein 47	up
22778	Ikzf1	IKAROS family zinc finger 1	up
16154	Il10ra	interleukin 10 receptor, alpha	up
16170	Il16	interleukin 16	up
16407	Itgae	integrin alpha E, epithelial-associated	up
16421	Itgb7	integrin beta 7	up
16428	Itk	IL2-inducible T-cell kinase	up
16498	Kcnab2	potassium voltage-gated channel, shaker-related subfamily, beta member 2	up
239743	Klhl6	kelch-like 6 (Drosophila)	up
259277	Klk8	kallikrein related-peptidase 8	up
16643	Klrd1	killer cell lectin-like receptor, subfamily D, member 1	up
16792	Laptm5	lysosomal-associated protein transmembrane 5	up
16797	Lat	linker for activation of T cells	up
56743	Lat2	linker for activation of T cells family, member 2	up
16818	Lck	lymphocyte protein tyrosine kinase	up
18826	Lcp1	lymphocyte cytosolic protein 1	up
107321	Lpxn	leupaxin	up
16985	Lsp1	lymphocyte specific 1	up
16994	Ltb	lymphotoxin B	up
17084	Ly86	lymphocyte antigen 86	up
17085	Ly9	lymphocyte antigen 9	up
17110	Lyz1	lysozyme 1	up
17105	Lyz2	lysozyme 2	up
17259	Mef2b	myocyte enhancer factor 2B	up
17304	Mfge8	milk fat globule-EGF factor 8 protein	up
17698	Msn	moesin	up
246177	Myo1g	myosin IG	up
16541	Napsa	napsin A aspartic peptidase	up
17972	Ncf4	neutrophil cytosolic factor 4	up
105855	Nckap1l	NCK associated protein 1 like	up
243910	Nfkbid	nuclear factor of kappa light polypeptide gene enhancer in B-cells inhibitor, delta	up

72310	Nkg7	natural killer cell group 7 sequence	up
434341	Nlrc5	NLR family, CARD domain containing 5	up
70113	Odf3b	outer dense fiber of sperm tails 3B	down
74191	P2ry13	purinergic receptor P2Y, G-protein coupled 13	up
239827	Pigz	phosphatidylinositol glycan anchor biosynthesis, class Z	up
18985	Pou2af1	POU domain, class 2, associating factor 1	up
67905	Ppm1m	protein phosphatase 1M	up
18751	Prkcb	protein kinase C, beta	up
16913	Psmb8	proteasome (prosome, macropain) subunit, beta type 8 (large multifunctional peptidase 7)	up
19245	Ptp4a3	protein tyrosine phosphatase 4a3	up
19265	Ptprcap	protein tyrosine phosphatase, receptor type, C polypeptide-associated protein	up
19354	Rac2	RAS-related C3 botulinum substrate 2	up
320484	Rasal3	RAS protein activator like 3	up
19419	Rasgrp1	RAS guanyl releasing protein 1	up
215653	Rassf2	Ras association (RalGDS/AF-6) domain family member 2	up
54354	Rassf5	Ras association (RalGDS/AF-6) domain family member 5	up
76438	Rftn1	raftlin lipid raft linker 1	up
78416	Rnase6	ribonuclease, RNase A family, 6	up
74131	Sash3	SAM and SH3 domain containing 3	up
20230	Satb1	special AT-rich sequence binding protein 1	up
20345	Selplg	selectin, platelet (p-selectin) ligand	up
20715	Serpina3g	serine (or cysteine) peptidase inhibitor, clade A, member 3G	up
546546	Serpina3h	serine (or cysteine) peptidase inhibitor, clade A, member 3H	up
27371	Sh2d2a	SH2 domain protein 2A	up
58194	Sh3kbp1	SH3-domain kinase binding protein 1	up
20491	Sla	src-like adaptor	up
227659	Slc2a6	solute carrier family 2 (facilitated glucose transporter), member 6	up
68682	Slc44a2	solute carrier family 44, member 2	up
64454	Slc5a4b	solute carrier family 5 (neutral amino acid transporters, system A), member 4b	down
50934	Slc7a8	solute carrier family 7 (cationic amino acid transporter, y+ system), member 8	down
71733	Susd2	sushi domain containing 2	down
21354	Tap1	transporter 1, ATP-binding cassette, sub-family B (MDR/TAP)	up
21838	Thy1	thymus cell antigen 1, theta	up
24099	Tnfsf13b	tumor necrosis factor (ligand) superfamily, member 13b	up
241732	Tspyl3	TSPY-like 3	up
24108	Ubd	ubiquitin D	up
22271	Upp1	uridine phosphorylase 1	up
22324	Vav1	vav 1 oncogene	up
22329	Vcam1	vascular cell adhesion molecule 1	up
22376	Was	Wiskott-Aldrich syndrome homolog (human)	up
22637	Zap70	zeta-chain (TCR) associated protein kinase	up

Supplementary Table S3

Differentialy expressed genes (|logFC|≥1; q < 0.05) in the colon mucosa of CR and monoassociated (N or O) vs. GF mice (contrast: CR/N/O vs. GF)

ENTREZ No.	Symbol	Gene name	Change
11668	Aldh1a1	aldehyde dehydrogenase family 1, subfamily A1	down
12424	Cck	cholecystokinin	down
16149	Cd74	CD74 antigen (invariant polypeptide of major histocompatibility complex, class II antigen-associated)	up
76279	Cyp2d26	cytochrome P450, family 2, subfamily d, polypeptide 26	down
14282	Fosb	FBJ osteosarcoma oncogene B	down
14537	Gcnt1	glucosaminyl (N-acetyl) transferase 1, core 2	up
14961	H2-Ab1	histocompatibility 2, class II antigen A, beta 1	up
14968	H2-Ea-ps	histocompatibility 2, class II antigen E alpha, pseudogene	up
67709	Reg4	regenerating islet-derived family, member 4	up
319848	Slc17a4	solute carrier family 17 (sodium phosphate), member 4	down
21888	Tle4	transducin-like enhancer of split 4, homolog of Drosophila E(spl)	down
54367	Zfp326	zinc finger protein 326	down

Supplementary Table S3

Differentialy expressed genes (|logFC|≥1; q < 0.05) in the mucosa of the middle part of the small intestine of conventionally reared (CR) and monoassociated (N or O) vs. germ-free (GF) mice (contrast: CR/N/O vs. GF)

ENTREZ No.	Symbol	Gene name	Change
58860	Adamdec1	ADAM-like, decysin 1	up
11816	Apoe	apolipoprotein E	up
14229	Fkbp5*	FK506 binding protein 5	C_GF: down<br>N/O_GF: up
15220	Foxq1	forkhead box Q1	down
384783	Irs2*	insulin receptor substrate 2	down
110454	Ly6a	lymphocyte antigen 6 complex, locus A	up
17384	Mmp10	matrix metallopeptidase 10	up
18089	Nkx2-3	NK2 transcription factor related, locus 3 (Drosophila)	up
84094	Plvap	plasmalemma vesicle associated protein	up
21813	Tgfbr2	transforming growth factor, beta receptor II	up

*) these genes did not pass the significance criterion ($p < 0.05$) in the comparison O vs. GF; by other genes the p value was < 0.05 in all three contrasts (i.e. CR vs. GF; N vs. GF; O vs. GF)

Supplementary Table S3

Differentialy expressed genes (|logFC|≥1; q < 0.05) in the mucosa of the distal part of the small intestine of CR and monoassociated (N or O) vs. GF mice (contrast: CR/N/O vs. GF)

ENTREZ No.	Symbol	Gene name	Change
11816	Apoe	apolipoprotein E	up
65256	Asb2	ankyrin repeat and SOCS box-containing 2	up
12111	Bgn	biglycan	up
11537	Cfd	complement factor D (adipsin)	C_GF: down<br>N/O_GF: up
13614	Edn1	endothelin 1	down
384783	Irs2	insulin receptor substrate 2	down
18387	Oprk1	opioid receptor, kappa 1	down
20568	Slpi	secretory leukocyte peptidase inhibitor	up
21813	Tgfbr2	transforming growth factor, beta receptor II	up

Supplementary Table S4

Normalized CT values in the qRT-PCR analysis of genes differentialy expressed in colonorganoids of CR vs. GF mice (contrast: CR vs. GF)
Corresponding diagram is given in Fig. 4C.

Symbol	CR		GF	
Ascl2	27.93	27.84	29.15	29.52
	27.29	28.04	29.25	29.45
Lgr5	27.10	27.69	28.63	28.66
	26.91	27.29	28.22	27.92
Krt13	32.73	32.33	29.81	30.03
	32.65	32.72	29.30	30.02
Krt14	29.50	29.61	26.79	26.34
	29.73	29.00	26.56	26.66
Oc2	32.63	32.72	28.52	28.23
	32.89	32.72	28.76	28.86
Wnt7a	32.29	31.98	28.12	28.61
	31.78	32.50	28.54	28.28
Wnt7b	30.74	31.72	27.50	27.66
	30.93	31.45	27.67	28.02

O

Supplementary Table S4

Normaliuzed CT values obtained by qRT-PCR analysis of genes differentialy expressed in the mucosa of the middle part of the small intestine of CR, N and O vs. GF mice (contrast: CR/N/O vs. GF) Corresponding diagram is given in Fig. 2B.

Symbol	CR		N		O		GF	
Adamdec1	25.18	25.43	27.19	27.64	27.96	26.57	28.47	28.00
	25.29	26.52	27.52	26.93	27.49	27.78	28.57	28.05
Apoe	23.94	24.32	25.96	25.56	26.10	25.93	25.76	26.01
	23.78	24.55	26.09	26.05	25.41	25.92	25.73	26.24
Fkbp5	29.05	30.43	25.77	25.48	26.72	26.95	26.66	26.60
	30.15	29.13	25.48	25.54	26.20	26.95	27.43	27.21
Foxq1	29.64	29.84	29.60	28.79	29.82	29.89	28.20	28.34
	30.07	29.94	28.75	29.20	29.56	30.01	27.85	28.53
Irs2	27.72	28.37	27.57	28.34	27.52	27.52	25.94	26.07
	27.55	27.36	27.23	28.85	27.73	26.08	25.67	25.79
Ly6a	26.25	26.05	28.82	28.61	28.47	28.07	30.03	30.34
	26.42	26.39	28.97	28.58	27.58	28.60	29.28	29.05
Mmp10	27.15	27.89	28.81	28.56	29.03	28.14	30.49	29.14
	27.15	27.21	29.14	28.92	28.52	28.31	30.01	29.64
Nkx2-3	30.94	30.33	31.91	31.91	32.00	31.36	32.09	32.73
	30.11	29.82	32.10	31.94	31.31	32.03	32.03	31.34
Plvap	27.03	26.65	28.89	28.35	28.74	28.45	29.81	30.13
	27.45	27.13	28.70	28.62	28.88	28.47	29.52	29.72
Tgfbr2	30.56	31.05	32.51	32.76	33.66	33.04	34.17	33.57
	31.51	31.66	33.16	33.73	32.55	33.03	33.81	34.27

Supplementary Table S4

CT values obtained by qRT-PCR analysis of genes differentialy expressed in the mucosa of the distal part of the small intestine of CR, N and O vs. GF mice (contrast: CR/N/O vs. GF)
Corresponding diagram is given in Fig. 2B.

Symbol	CR		N		O		GF	
Apoe	23.10	23.34	25.03	24.95	24.30	25.66	26.86	26.99
	23.10	22.93	24.49	24.73	24.94	24.90	26.35	26.99
Asb2	26.57	26.22	27.17	27.74	28.20	29.06	29.84	30.90
	26.71	26.36	27.40	28.12	28.25	29.10	29.69	30.76
Bgn	25.33	26.27	26.98	27.34	27.15	27.03	29.78	29.86
	26.31	25.31	26.41	26.94	27.09	26.44	28.58	29.10
Edn1	28.85	28.81	26.95	27.99	29.25	30.26	26.87	26.97
	29.53	29.21	27.14	27.63	30.57	29.36	26.84	26.79
Irs2	25.98	26.28	28.89	28.04	27.88	27.10	25.46	25.06
	26.30	26.22	28.32	28.26	27.82	27.80	24.90	25.02
Oprk1	28.10	28.83	25.66	25.21	25.57	26.33	25.38	25.62
	29.11	28.96	25.64	25.79	25.30	26.10	25.24	25.07
Slpi	28.60	28.26	26.10	27.40	27.08	27.35	31.61	32.17
	28.94	27.88	26.02	27.61	26.74	26.75	30.69	31.30
Tgfbr2	27.90	27.78	28.14	28.30	28.83	28.43	33.09	32.55
	27.27	27.37	29.23	28.29	29.26	28.85	32.10	32.22

Supplementary Table S4

CT values obtained by qRT-PCR analysis of genes differentialy expressed in the colonic mucosa of the small intestine of CR, N and O vs. GF mice (contrast: CR/N/O vs. GF)
Corresponding diagram is given in Fig. 3B.

Symbol	CR		N		O		GF	
Aldh1a1	31.97	32.53	30.51	31.46	31.85	31.57	29.23	30.43
	32.04	31.84	31.81	31.73	32.71	32.66	29.67	28.91
Cck	28.41	29.09	31.32	30.42	30.22	29.68	26.25	26.84
	28.38	29.25	31.46	30.37	30.45	30.48	25.53	25.37
Cd74	22.89	22.40	23.78	23.92	23.41	23.98	27.16	25.98
	23.37	22.47	24.70	24.38	24.91	24.79	27.16	27.05
Cyp2d26	26.02	26.33	25.21	25.27	24.98	25.06	23.28	23.99
	26.03	26.23	25.82	25.35	25.39	25.88	24.48	23.66
Fosb	30.16	29.80	32.26	33.32	30.85	30.71	28.31	28.01
	30.38	31.00	32.75	32.92	31.91	31.13	29.35	29.07
Gcnt1	24.51	24.39	25.00	24.72	24.75	25.53	25.72	25.31
	24.13	24.15	25.49	25.30	25.03	25.36	25.45	25.47
H2-Ab1	21.56	22.18	22.48	22.62	22.08	23.25	25.62	24.67
	21.72	21.26	22.96	23.16	23.51	22.87	25.68	25.31
H2-Ea-ps	22.61	21.29	22.49	22.35	22.39	23.10	24.58	24.94
	22.01	22.15	22.94	22.80	23.67	23.37	25.66	25.30
Reg4	19.75	19.29	21.61	21.35	19.51	20.90	21.39	22.19
	19.88	20.78	22.10	21.65	21.37	20.87	22.15	22.28
Slc17a4	26.91	26.12	27.20	26.89	26.30	25.96	24.61	24.58
	26.56	26.30	26.70	26.48	25.78	26.16	24.94	24.90
Tle4	26.85	26.34	25.82	25.83	25.24	25.49	25.55	24.98
	26.02	26.39	26.23	26.14	25.50	26.03	25.01	25.46
Zfp326	27.94	26.83	26.76	26.06	26.35	26.32	25.01	25.51
	27.94	26.83	26.22	26.87	26.25	26.28	25.85	25.85

Supplementary Table S5

Differentialy expressed genes (|logFC|≥1; q < 0.05) in the mucosa of the middle part of the small intestine in monoassociated mice (contrast: N_O)

ENTREZ No.	Symbol	Gene name	Log FC
78925	Srd5a1	steroid 5 alpha-reductase 1	-1.13
217721	Mfsd7c	major facilitator superfamily domain containing 7C	-1.37
20531	Slc34a2	solute carrier family 34 (sodium phosphate), member 2	-2.29

Supplementary Table S5

Differentialy expressed genes (|logFC|≥1; q < 0.05) in the colonic mucosa in monoassociated mice (contrast: N_O)

ENTREZ No.	Symbol	Gene name	Log FC
12350	Car3	carbonic anhydrase 3	3.97
13106	Cyp2e1	cytochrome P450, family 2, subfamily e, polypeptide 1	3.59
57435	Plin4	perilipin 4	3.48
11450	Adipoq	adiponectin, C1Q and collagen domain containing	3.45
13346	Des	desmin	3.02
18947	Pnliprp2	pancreatic lipase-related protein 2	2.97
14311	Cidec	cell death-inducing DFFA-like effector c	2.78
20249	Scd1	stearoyl-Coenzyme A desaturase 1	2.75
11754	Aoc3	amine oxidase, copper containing 3	2.64
22004	Tpm2	tropomyosin 2, beta	2.53
109791	Clps	colipase, pancreatic	2.43
16667	Krt17	keratin 17	2.31
22353	Vip	vasoactive intestinal polypeptide	2.26
29856	Smtn	smoothelin	2.19
80888	Hspb8	heat shock protein 8	2.14
12308	Calb2	calbindin 2	2.01
12509	Cd59a	CD59a antigen	1.97
17901	Myl1	myosin, light polypeptide 1	1.97
233335	Synm	synemin, intermediate filament protein	1.93
225341	Lims2	LIM and senescent cell antigen like domains 2	1.92
21826	Thbs2	thrombospondin 2	1.84
107589	Mylk	myosin, light polypeptide kinase	1.83
17984	Ndn	necdin	1.81
57262	Retnla	resistin like alpha	1.81
246747	Adig	adipogenin	1.79

15439	Hp	haptoglobin	1.77
16846	Lep	leptin	1.76
19242	Ptn	pleiotrophin	1.76
18405	Orm1	orosomucoid 1	1.73
13007	Csrp1	cysteine and glycine-rich protein 1	1.71
14560	Gdf10	growth differentiation factor 10	1.67
74116	Pi16	peptidase inhibitor 16	1.60
29859	Sult4a1	sulfotransferase family 4A, member 1	1.58
59095	Fxyd6	FXYD domain-containing ion transport regulator 6	1.56
67896	Ccdc80	coiled-coil domain containing 80	1.55
18113	Nnmt	nicotinamide N-methyltransferase	1.53
13640	Efna5	ephrin A5	1.52
93689	Lmod1	leiomodin 1 (smooth muscle)	1.52
654812	Angptl7	angiopoietin-like 7	1.50
77037	Mrap	melanocortin 2 receptor accessory protein	1.50
70784	Rasl12	RAS-like, family 12	1.47
13807	Eno2	enolase 2, gamma neuronal	1.43
21835	Thrsp	thyroid hormone responsive SPOT14 homolog (Rattus)	1.42
76905	Lrg1	leucine-rich alpha-2-glycoprotein 1	1.39
20618	Sncg	synuclein, gamma	1.37
20257	Stmn2	stathmin-like 2	1.36
12889	Cplx1	complexin 1	1.34
67399	Pdlim7	PDZ and LIM domain 7	1.33
243912	Hspb6	heat shock protein, alpha-crystallin-related, B6	1.32
13628	Eef1a2	eukaryotic translation elongation factor 1 alpha 2	1.31
99326	Garnl3	GTPase activating RANGAP domain-like 3	1.29
14261	Fmo1	flavin containing monooxygenase 1	1.24
225642	Grp	gastrin releasing peptide	1.22
244058	Rgma	RGM domain family, member A	1.20
23948	Mmp17	matrix metallopeptidase 17	1.18
53376	Usp2	ubiquitin specific peptidase 2	1.18
68939	Rasl11b	RAS-like, family 11, member B	1.17
52906	Ahi1	Abelson helper integration site 1	1.15

20411	Sorbs1	sorbin and SH3 domain containing 1	1.13
22223	Uchl1	ubiquitin carboxy-terminal hydrolase L1	1.13
73712	Dmkn	dermokine	1.11
20254	Scg2	secretogranin II	1.11
268534	Sntg2	syntrophin, gamma 2	1.11
14165	Fgf10	fibroblast growth factor 10	1.10
83767	Wasf1	WASP family 1	1.09
244757	Glb1l2	galactosidase, beta 1-like 2	1.07
27276	Plekhb1	pleckstrin homology domain containing, family B (evectins) member 1	1.05
19762	Rit2	Ras-like without CAAX 2	1.01
12096	Bglap	bone gamma carboxyglutamate protein	1.00

Supplementary Table S6

Differentialy expressed genes (|logFC|≥1; q < 0.05) in the colon organoids of GF and monoassociated (N or O) vs. CR mice (contrast: GF/N/O vs. CR)

ENTREZ No.	Symbol	Gene name	Change
26364	Adgre5	adhesion G protein-coupled receptor E5	up
11670	Aldh3a1	aldehyde dehydrogenase family 3, subfamily A1	up
107765	Ankrd1	ankyrin repeat domain 1 (cardiac muscle)	up
11752	Anxa8	annexin A8	up
229672	Bcl2l15	BCLl2-like 15	up
83675	Bicc1	BicC family RNA binding protein 1	up
12263	C2	complement component 2 (within H-2S)	up
56078	Car5b	carbonic anhydrase 5b, mitochondrial	up
104252	Cdc42ep2	CDC42 effector protein (Rho GTPase binding) 2	up
12554	Cdh13	cadherin 13	up
12556	Cdh16	cadherin 16	up
12579	Cdkn2b	cyclin-dependent kinase inhibitor 2B (p15, inhibits CDK4)	up
80982	Cemip	cell migration inducing protein, hyaluronan binding	up
72361	Ces2g	carboxylesterase 2G	up
69454	Clic3	chloride intracellular channel 3	up
12759	Clu	clusterin	up
12822	Col18a1	collagen, type XVIII, alpha 1	up
13034	Ctse	cathepsin E	up
231293	Cwh43	cell wall biogenesis 43 C-terminal homolog	up
56448	Cyp2d22	cytochrome P450, family 2, subfamily d, polypeptide 22	up
67859	Cysrt1	cysteine rich tail 1	up
13363	Dhh	desert hedgehog	up
13848	Ephb6	Eph receptor B6	up
14062	F2r	coagulation factor II (thrombin) receptor	up
70564	Fam213a	family with sequence similarity 213, member A	up
230904	Fbxo2	F-box protein 2	up
14221	Fjx1	four jointed box 1 (Drosophila)	up
68794	Flnc	filamin C, gamma	up
108017	Fxyd4	FXYD domain-containing ion transport regulator 4	up
104174	Gldc	glycine decarboxylase	up
14858	Gsta2	glutathione S-transferase, alpha 2 (Yc2)	up
14859	Gsta3	glutathione S-transferase, alpha 3	up
15415	Hoxb7	homeobox B7	up
29817	Igfbp7	insulin-like growth factor binding protein 7	up
76527	Il34	interleukin 34	up
16447	Ivl	involucrin	up
317652	Klk15	kallikrein related-peptidase 15	up
16663	Krt13	keratin 13	up
16664	Krt14	keratin 14	up
16682	Krt4	keratin 4	up
16688	Krt6b	keratin 6B	up
16680	Krt84	keratin 84	up
239673	Krt90	keratin 90	up
16774	Lama3	laminin, alpha 3	up
16782	Lamc2	laminin, gamma 2	up
107753	Lgals2	lectin, galactose-binding, soluble 2	up
67573	Loxl4	lysyl oxidase-like 4	up
17153	Mal	myelin and lymphocyte protein, T cell differentiation protein	up
17436	Me1	malic enzyme 1, NADP(+)-dependent, cytosolic	up
71306	Mfap3l	microfibrillar-associated protein 3-like	up
17524	Mpp1	membrane protein, palmitoylated	up

56047	Msln	mesothelin	up
17119	Mxd1	MAX dimerization protein 1	up
17965	Nbl1	neuroblastoma, suppression of tumorigenicity 1	up
18104	Nqo1	NAD(P)H dehydrogenase, quinone 1	up
23959	Nt5e	5' nucleotidase, ecto	up
225631	Onecut2	one cut domain, family member 2	up
18599	Padi1	peptidyl arginine deiminase, type I	up
210622	Pamr1	peptidase domain containing associated with muscle regeneration 1	up
236899	Pcyt1b	phosphate cytidylyltransferase 1, choline, beta isoform	up
73182	Pear1	platelet endothelial aggregation receptor 1	up
18645	Pfn2	profilin 2	up
18791	Plat	plasminogen activator, tissue	up
72469	Plcd3	phospholipase C, delta 3	up
18806	Pld2	phospholipase D2	up
109042	Prkcdbp	protein kinase C, delta binding protein	up
70835	Prss22	protease, serine 22	up
76072	Rnf183	ring finger protein 183	up
67860	S100a16	S100 calcium binding protein A16	up
20208	Saa1	serum amyloid A 1	up
26456	Sema4g	sema domain, immunoglobulin domain (Ig), transmembrane domain (TM) and short cytoplasmic domain, (semaphorin) 4G	up
103710	Slc35e4	solute carrier family 35, member E4	up
59033	Slc4a8	solute carrier family 4 (anion exchanger), member 8	up
108115	Slco4a1	solute carrier organic anion transporter family, member 4a1	up
20753	Sprr1a	small proline-rich protein 1A	up
211389	Suox	sulfite oxidase	up
55925	Syt8	synaptotagmin VIII	up
56370	Tagln3	transgelin 3	up
21826	Thbs2	thrombospondin 2	up
229302	Tm4sf4	transmembrane 4 superfamily member 4	up
21933	Tnfrsf10b	tumor necrosis factor receptor superfamily, member 10b	up
21953	Tnni2	troponin I, skeletal, fast 2	up
67866	Wfdc1	WAP four-disulfide core domain 1	up
22421	Wnt7a	wingless-type MMTV integration site family, member 7A	up
22422	Wnt7b	wingless-type MMTV integration site family, member 7B	up
58206	Zbtb32	zinc finger and BTB domain containing 32	up
207259	Zbtb7c	zinc finger and BTB domain containing 7C	up
11302	Aatk	apoptosis-associated tyrosine kinase	down
16365	Acod1	aconitate decarboxylase 1	down
16365	Acod1	aconitate decarboxylase 1	down
26897	Acot1	acyl-CoA thioesterase 1	down
11459	Acta1	actin, alpha 1, skeletal muscle	down
243277	Adgrd1	adhesion G protein-coupled receptor D1	down
13733	Adgre1	adhesion G protein-coupled receptor E1	down
11541	Adora2b	adenosine A2b receptor	down
381524	AI427809	expressed sequence AI427809	down
72535	Aldh1b1	aldehyde dehydrogenase 1 family, member B1	down
11690	Alox5ap	arachidonate 5-lipoxygenase activating protein	down
270152	Amica1	adhesion molecule, interacts with CXADR antigen 1	down
16790	Anpep	alanyl (membrane) aminopeptidase	down
27052	Aoah	acyloxyacyl hydrolase	down
11816	Apoe	apolipoprotein E	down
11829	Aqp4	aquaporin 4	down
106952	Arap3	ArfGAP with RhoGAP domain, ankyrin repeat and PH domain 3	down
11846	Arg1	arginase, liver	down
231532	Arhgap24	Rho GTPase activating protein 24	down
232201	Arhgap25	Rho GTPase activating protein 25	down
226652	Arhgap30	Rho GTPase activating protein 30	down

11857	Arhgdib	Rho, GDP dissociation inhibitor (GDI) beta	down
622434	Arhgef26	Rho guanine nucleotide exchange factor (GEF) 26	down
73341	Arhgef6	Rac/Cdc42 guanine nucleotide exchange factor (GEF) 6	down
219144	Arl11	ADP-ribosylation factor-like 11	down
74448	Arl13a	ADP-ribosylation factor-like 13A	down
17173	Ascl2	achaete-scute family bHLH transcription factor 2	down
232984	B3gnt8	UDP-GlcNAc:betaGal beta-1,3-N-acetylglucosaminyltransferase 8	down
12046	Bcl2a1c	B cell leukemia/lymphoma 2 related protein A1c	down
12229	Btk	Bruton agammaglobulinemia tyrosine kinase	down
12259	C1qa	complement component 1, q subcomponent, alpha polypeptide	down
12260	C1qb	complement component 1, q subcomponent, beta polypeptide	down
12262	C1qc	complement component 1, q subcomponent, C chain	down
12774	Ccr5	chemokine (C-C motif) receptor 5	down
68891	Cd177	CD177 antigen	down
12487	Cd28	CD28 antigen	down
246746	Cd300lf	CD300 molecule like family member F	down
12489	Cd33	CD33 antigen	down
23833	Cd52	CD52 antigen	down
12508	Cd53	CD53 antigen	down
12514	Cd68	CD68 antigen	down
12517	Cd72	CD72 antigen	down
12522	Cd83	CD83 antigen	down
12523	Cd84	CD84 antigen	down
12524	Cd86	CD86 antigen	down
17064	Cd93	CD93 antigen	down
18636	Cfp	complement factor properdin	down
12653	Chgb	chromogranin B	down
229927	Clca3b	chloride channel accessory 3B	down
17474	Clec4d	C-type lectin domain family 4, member d	down
56620	Clec4n	C-type lectin domain family 4, member n	down
56644	Clec7a	C-type lectin domain family 7, member a	down
380686	Cnrip1	cannabinoid receptor interacting protein 1	down
12721	Coro1a	coronin, actin binding protein 1A	down
12978	Csf1r	colony stimulating factor 1 receptor	down
13051	Cx3cr1	chemokine (C-X3-C motif) receptor 1	down
57266	Cxcl14	chemokine (C-X-C motif) ligand 14	down
12767	Cxcr4	chemokine (C-X-C motif) receptor 4	down
58861	Cysltr1	cysteinyl leukotriene receptor 1	down
72318	Cyth4	cytohesin 4	down
13132	Dab2	disabled 2, mitogen-responsive phosphoprotein	down
110197	Dgkg	diacylglycerol kinase, gamma	down
13417	Dnah8	dynein, axonemal, heavy chain 8	down
54427	Dnmt3l	DNA (cytosine-5-)-methyltransferase 3-like	down
210293	Dock10	dedicator of cytokinesis 10	down
319446	Dpep2	dipeptidase 2	down
71712	Dram1	DNA-damage regulated autophagy modulator 1	down
50498	Ebi3	Epstein-Barr virus induced gene 3	down
13723	Emb	embigin	down
246707	Emilin2	elastin microfibril interfacer 2	down
14017	Evi2a	ecotropic viral integration site 2a	down
14058	F10	coagulation factor X	down
74145	F13a1	coagulation factor XIII, A1 subunit	down
14077	Fabp3	fatty acid binding protein 3, muscle and heart	down
14130	Fcgr2b	Fc receptor, IgG, low affinity IIb	down
14131	Fcgr3	Fc receptor, IgG, low affinity III	down
246256	Fcgr4	Fc receptor, IgG, low affinity IV	down
14133	Fcna	ficolin A	down
80891	Fcrls	Fc receptor-like S, scavenger receptor	down

108101	Fermt3	fermitin family member 3	down
233079	Ffar2	free fatty acid receptor 2	down
100040736	Foxd2os	forkhead box D2, opposite strand	down
14289	Fpr2	formyl peptide receptor 2	down
18301	Fxyd5	FXYD domain-containing ion transport regulator 5	down
14457	Gas7	growth arrest specific 7	down
14526	Gcg	glucagon	down
384009	Glipr2	GLI pathogenesis-related 2	down
93695	Gpnmb	glycoprotein (transmembrane) nmb	down
80910	Gpr84	G protein-coupled receptor 84	down
14783	Grb10	growth factor receptor bound protein 10	down
268527	Greb1	gene regulated by estrogen in breast cancer protein	down
14990	H2-M2	histocompatibility 2, M region locus 2	down
15109	Hal	histidine ammonia lyase	down
15163	Hcls1	hematopoietic cell specific Lyn substrate 1	down
212032	Hk3	hexokinase 3	down
15439	Hp	haptoglobin	down
15559	Htr2b	5-hydroxytryptamine (serotonin) receptor 2B	down
74096	Hvcn1	hydrogen voltage-gated channel 1	down
226691	Ifi207	interferon activated gene 207	down
76933	Ifi27l2a	interferon, alpha-inducible protein 27 like 2A	down
68713	Ifitm1	interferon induced transmembrane protein 1	down
66141	Ifitm3	interferon induced transmembrane protein 3	down
16000	Igf1	insulin-like growth factor 1	down
16197	Il7r	interleukin 7 receptor	down
23919	Insl5	insulin-like 5	down
73914	Irak3	interleukin-1 receptor-associated kinase 3	down
27056	Irf5	interferon regulatory factor 5	down
16498	Kcnab2	potassium voltage-gated channel, shaker-related subfamily, beta member 2	down
239743	Klhl6	kelch-like 6	down
16792	Laptm5	lysosomal-associated protein transmembrane 5	down
56743	Lat2	linker for activation of T cells family, member 2	down
56743	Lat2	linker for activation of T cells family, member 2	down
18826	Lcp1	lymphocyte cytosolic protein 1	down
14160	Lgr5	leucine rich repeat containing G protein coupled receptor 5	down
16909	Lmo2	LIM domain only 2	down
107321	Lpxn	leupaxin	down
16985	Lsp1	lymphocyte specific 1	down
17084	Ly86	lymphocyte antigen 86	down
17085	Ly9	lymphocyte antigen 9	down
17110	Lyz1	lysozyme 1	down
17105	Lyz2	lysozyme 2	down
380732	Milr1	mast cell immunoglobulin like receptor 1	down
17381	Mmp12	matrix metallopeptidase 12	down
17395	Mmp9	matrix metallopeptidase 9	down
68774	Ms4a6d	membrane-spanning 4-domains, subfamily A, member 6D	down
109225	Ms4a7	membrane-spanning 4-domains, subfamily A, member 7	down
20288	Msr1	macrophage scavenger receptor 1	down
17912	Myo1b	myosin IB	down
17916	Myo1f	myosin IF	down
17972	Ncf4	neutrophil cytosolic factor 4	down
105855	Nckap1l	NCK associated protein 1 like	down
11925	Neurog3	neurogenin 3	down
237038	Nox1	NADPH oxidase 1	down
109648	Npy	neuropeptide Y	down
18166	Npy1r	neuropeptide Y receptor Y1	down
224109	Nrros	negative regulator of reactive oxygen species	down
78252	Nxpe2	neurexophilin and PC-esterase domain family, member 2	down

70155	Ogfrl1	opioid growth factor receptor-like 1	down
99543	Olfml3	olfactomedin-like 3	down
18413	Osm	oncostatin M	down
74191	P2ry13	purinergic receptor P2Y, G-protein coupled 13	down
140795	P2ry14	purinergic receptor P2Y, G-protein coupled, 14	down
56744	Pf4	platelet factor 4	down
98496	Pid1	phosphotyrosine interaction domain containing 1	down
231805	Pilra	paired immunoglobin-like type 2 receptor alpha	down
18780	Pla2g2a	phospholipase A2, group IIA (platelets, synovial fluid)	down
104759	Pld4	phospholipase D family, member 4	down
102595	Plekho2	pleckstrin homology domain containing, family O member 2	down
67784	Plxnd1	plexin D1	down
18947	Pnliprp2	pancreatic lipase-related protein 2	down
18751	Prkcb	protein kinase C, beta	down
353211	Prune2	prune homolog 2	down
19227	Pthlh	parathyroid hormone-like peptide	down
19277	Ptpro	protein tyrosine phosphatase, receptor type, O	down
110095	Pygl	liver glycogen phosphorylase	down
217212	Pyy	peptide YY	down
213391	Rassf4	Ras association (RalGDS/AF-6) domain family member 4	down
76438	Rftn1	raftlin lipid raft linker 1	down
66214	Rgcc	regulator of cell cycle	down
50778	Rgs1	regulator of G-protein signaling 1	down
93726	Rnase2a	ribonuclease, RNase A family, 2A (liver, eosinophil-derived neurotoxin)	down
20201	S100a8	S100 calcium binding protein A8 (calgranulin A)	down
67742	Samsn1	SAM domain, SH3 domain and nuclear localization signals, 1	down
72821	Scn2b	sodium channel, voltage-gated, type II, beta	down
20970	Sdc3	syndecan 3	down
20345	Selplg	selectin, platelet (p-selectin) ligand	down
320007	Sidt1	SID1 transmembrane family, member 1	down
20612	Siglec1	sialic acid binding Ig-like lectin 1, sialoadhesin	down
19261	Sirpa	signal-regulatory protein alpha	down
20491	Sla	src-like adaptor	down
18173	Slc11a1	solute carrier family 11 (proton-coupled divalent metal ion transporters), member 1	down
171286	Slc12a8	solute carrier family 12 (potassium/chloride transporters), member 8	down
65221	Slc15a3	solute carrier family 15, member 3	down
105727	Slc38a1	solute carrier family 38, member 1	down
67547	Slc39a8	solute carrier family 39 (metal ion transporter), member 8	down
14411	Slc6a12	solute carrier family 6 (neurotransmitter transporter, betaine/GABA), member 12	down
11988	Slc7a2	solute carrier family 7 (cationic amino acid transporter, y+ system), member 2	down
50934	Slc7a8	solute carrier family 7 (cationic amino acid transporter, y+ system), member 8	down
20555	Slfn1	schlafen 1	down
20568	Slpi	secretory leukocyte peptidase inhibitor	down
20446	St6galnac2	ST6 (alpha-N-acetyl-neuraminyl-2,3-beta-galactosyl-1,3)-N-acetylgalactosaminide alpha-2,6-sialyltransferase 2	down
192187	Stab1	stabilin 1	down
236920	Stard8	START domain containing 8	down
21391	Tbxas1	thromboxane A synthase 1, platelet	down
21810	Tgfbi	transforming growth factor, beta induced	down
279572	Tlr13	toll-like receptor 13	down
170743	Tlr7	toll-like receptor 7	down
208213	Tmem132c	transmembrane protein 132C	down
252838	Tox	thymocyte selection-associated high mobility group box	down
83433	Trem2	triggering receptor expressed on myeloid cells 2	down

22368	Trpv2	transient receptor potential cation channel, subfamily V, member 2	down
22177	Tyrobp	TYRO protein tyrosine kinase binding protein	down
53376	Usp2	ubiquitin specific peptidase 2	down
57257	Vav3	vav 3 oncogene	down
22352	Vim	vimentin	down
22371	Vwf	Von Willebrand factor	down
22376	Was	Wiskott-Aldrich syndrome	down
77254	Yif1b	Yip1 interacting factor homolog B (S. cerevisiae)	down
71918	Zcchc24	zinc finger, CCHC domain containing 24	down

Supplementary Table S6

Differentialy expressed genes (|logFC|≥1; q < 0.05) in the colon organoids of GF vs. CR mice (contrast: GF vs. CR)

ENTREZ No.	Symbol	Gene name	Log FC
67866	Wfdc1	WAP four-disulfide core domain 1	3.94
83675	Bicc1	BicC family RNA binding protein 1	3.54
83675	Bicc1	BicC family RNA binding protein 1	3.41
225631	Onecut2	one cut domain, family member 2	3.29
18599	Padi1	peptidyl arginine deiminase, type I	3.19
29817	Igfbp7	insulin-like growth factor binding protein 7	3.17
225631	Onecut2	one cut domain, family member 2	3.11
107765	Ankrd1	ankyrin repeat domain 1 (cardiac muscle)	2.79
18791	Plat	plasminogen activator, tissue	2.71
16680	Krt84	keratin 84	2.68
317652	Klk15	kallikrein related-peptidase 15	2.61
67866	Wfdc1	WAP four-disulfide core domain 1	2.5
17153	Mal	myelin and lymphocyte protein, T cell differentiation protein	2.44
22421	Wnt7a	wingless-type MMTV integration site family, member 7A	2.4
22422	Wnt7b	wingless-type MMTV integration site family, member 7B	2.38
67866	Wfdc1	WAP four-disulfide core domain 1	2.27
17153	Mal	myelin and lymphocyte protein, T cell differentiation protein	2.22
229672	Bcl2l15	BCLl2-like 15	2.21
12822	Col18a1	collagen, type XVIII, alpha 1	2.15
20753	Sprr1a	small proline-rich protein 1A	2.14
16663	Krt13	keratin 13	2.1
236899	Pcyt1b	phosphate cytidylyltransferase 1, choline, beta isoform	2.07
16664	Krt14	keratin 14	2.07
17965	Nbl1	neuroblastoma, suppression of tumorigenicity 1	2.04
108017	Fxyd4	FXYD domain-containing ion transport regulator 4	2.04
108017	Fxyd4	FXYD domain-containing ion transport regulator 4	2.03
12876	Cpe	carboxypeptidase E	2.03
14221	Fjx1	four jointed box 1 (Drosophila)	2.02
56448	Cyp2d22	cytochrome P450, family 2, subfamily d, polypeptide 22	2.01
80982	Cemip	cell migration inducing protein, hyaluronan binding	2.01
69454	Clic3	chloride intracellular channel 3	2
22422	Wnt7b	wingless-type MMTV integration site family, member 7B	1.97
20897	Stra6	stimulated by retinoic acid gene 6	1.97
17436	Me1	malic enzyme 1, NADP(+)-dependent, cytosolic	1.89
70835	Prss22	protease, serine 22	1.84
68794	Flnc	filamin C, gamma	1.83
16774	Lama3	laminin, alpha 3	1.82
17965	Nbl1	neuroblastoma, suppression of tumorigenicity 1	1.81
23959	Nt5e	5' nucleotidase, ecto	1.78
21953	Tnni2	troponin I, skeletal, fast 2	1.77
18645	Pfn2	profilin 2	1.76
56370	Tagln3	transgelin 3	1.75
16688	Krt6b	keratin 6B	1.75
104174	Gldc	glycine decarboxylase	1.75
107753	Lgals2	lectin, galactose-binding, soluble 2	1.74
13363	Dhh	desert hedgehog	1.74
18645	Pfn2	profilin 2	1.73
21923	Tnc	tenascin C	1.67
14857	Gsta1	Mus musculus glutathione S-transferase, alpha 1 (Ya) (Gsta1), mRNA.	1.67
12554	Cdh13	cadherin 13	1.64

19142	Prss12	protease, serine 12 neurotrypsin (motopsin)	1.63
382643	Ahnak2	Mus musculus AHNAK nucleoprotein 2 (Ahnak2), mRNA.	1.63
17436	Me1	malic enzyme 1, NADP(+)-dependent, cytosolic	1.61
12759	Clu	clusterin	1.6
79202	Tnfrsf22	tumor necrosis factor receptor superfamily, member 22	1.58
14858	Gsta2	glutathione S-transferase, alpha 2 (Yc2)	1.58
13848	Ephb6	Eph receptor B6	1.57
16560	Kif1a	kinesin family member 1A	1.56
13370	Dio1	deiodinase, iodothyronine, type I	1.56
114301	Palmd	palmdelphin	1.55
107765	Ankrd1	ankyrin repeat domain 1 (cardiac muscle)	1.54
70564	Fam213a	family with sequence similarity 213, member A	1.54
55925	Syt8	synaptotagmin VIII	1.53
13034	Ctse	cathepsin E	1.53
14219	Ctgf	connective tissue growth factor	1.53
238055	Apob	apolipoprotein B	1.53
73182	Pear1	platelet endothelial aggregation receptor 1	1.52
19142	Prss12	protease, serine 12 neurotrypsin (motopsin)	1.51
13370	Dio1	deiodinase, iodothyronine, type I	1.5
11752	Anxa8	annexin A8	1.48
17119	Mxd1	MAX dimerization protein 1	1.46
74253	Klrg2	killer cell lectin-like receptor subfamily G, member 2	1.44
66938	Sh3d21	SH3 domain containing 21	1.44
16782	Lamc2	laminin, gamma 2	1.43
56047	Msln	mesothelin	1.42
104252	Cdc42ep2	CDC42 effector protein (Rho GTPase binding) 2	1.39
16682	Krt4	keratin 4	1.38
192212	Prom2	prominin 2	1.37
56533	Rgs17	regulator of G-protein signaling 17	1.36
231293	Cwh43	cell wall biogenesis 43 C-terminal homolog	1.36
210622	Pamr1	peptidase domain containing associated with muscle regeneration 1	1.35
18806	Pld2	phospholipase D2	1.34
70564	Fam213a	family with sequence similarity 213, member A	1.34
73569	Vgll3	vestigial like family member 3	1.33
67859	Cysrt1	cysteine rich tail 1	1.33
71306	Mfap3l	microfibrillar-associated protein 3-like	1.32
16174	Il18rap	interleukin 18 receptor accessory protein	1.32
268860	Abat	4-aminobutyrate aminotransferase	1.31
23959	Nt5e	5' nucleotidase, ecto	1.3
12153	Bmp1	bone morphogenetic protein 1	1.3
211389	Suox	sulfite oxidase	1.28
59033	Slc4a8	solute carrier family 4 (anion exchanger), member 8	1.27
73182	Pear1	platelet endothelial aggregation receptor 1	1.27
94242	Tinagl1	tubulointerstitial nephritis antigen-like 1	1.26
53897	Gal3st1	galactose-3-O-sulfotransferase 1	1.26
21987	Tpd52l1	tumor protein D52-like 1	1.25
52466	Slc46a1	solute carrier family 46, member 1	1.24
320415	Gchfr	GTP cyclohydrolase I feedback regulator	1.24
14187	Akr1b8	aldo-keto reductase family 1, member B8	1.24
77827	Krba1	KRAB-A domain containing 1	1.23
94242	Tinagl1	tubulointerstitial nephritis antigen-like 1	1.22
74090	Paqr5	progestin and adipoQ receptor family member V	1.22
231293	Cwh43	cell wall biogenesis 43 C-terminal homolog	1.22
109042	Prkcdbp	protein kinase C, delta binding protein	1.21
69195	Tmem121	transmembrane protein 121	1.2
12153	Bmp1	bone morphogenetic protein 1	1.2
56847	Aldh1a3	aldehyde dehydrogenase family 1, subfamily A3	1.2

22409	Wnt10a	wingless-type MMTV integration site family, member 10A	1.19
83767	Wasf1	WAS protein family, member 1	1.19
17524	Mpp1	membrane protein, palmitoylated	1.19
15460	Hr	hairless	1.19
56078	Car5b	carbonic anhydrase 5b, mitochondrial	1.19
71306	Mfap3l	microfibrillar-associated protein 3-like	1.19
67860	S100a16	S100 calcium binding protein A16	1.18
218952	Fermt2	fermitin family member 2	1.18
12153	Bmp1	bone morphogenetic protein 1	1.18
57875	Angptl4	angiopoietin-like 4	1.18
207259	Zbtb7c	zinc finger and BTB domain containing 7C	1.17
114255	Dok4	docking protein 4	1.17
11752	Anxa8	annexin A8	1.17
109042	Prkcdbp	protein kinase C, delta binding protein	1.16
93672	Il24	interleukin 24	1.16
269356	Slc4a11	solute carrier family 4, sodium bicarbonate transporter-like, member 11	1.15
72469	Plcd3	phospholipase C, delta 3	1.15
12332	Capg	capping protein (actin filament), gelsolin-like	1.15
230752	Eva1b	eva-1 homolog B (C. elegans)	1.15
231147	Sh3tc1	SH3 domain and tetratricopeptide repeats 1	1.14
19659	Rbp1	retinol binding protein 1, cellular	1.14
13036	Ctsh	cathepsin H	1.14
57278	Bcam	basal cell adhesion molecule	1.14
94180	Acsbg1	acyl-CoA synthetase bubblegum family member 1	1.14
58206	Zbtb32	zinc finger and BTB domain containing 32	1.13
13821	Epb41l1	erythrocyte membrane protein band 4.1 like 1	1.13
76527	Il34	interleukin 34	1.13
76943	Psapl1	prosaposin-like 1	1.12
77827	Krba1	KRAB-A domain containing 1	1.12
67603	Dusp6	dual specificity phosphatase 6	1.12
13036	Ctsh	cathepsin H	1.12
56708	Clcf1	cardiotrophin-like cytokine factor 1	1.12
12579	Cdkn2b	cyclin-dependent kinase inhibitor 2B (p15, inhibits CDK4)	1.12
17760	Map6	microtubule-associated protein 6	1.11
320415	Gchfr	GTP cyclohydrolase I feedback regulator	1.11
13821	Epb41l1	erythrocyte membrane protein band 4.1 like 1	1.11
14027	Evpl	envoplakin	1.1
11603	Agrn	agrin	1.09
68178	Cgnl1	cingulin-like 1	1.09
71907	Serpina9	cysteine) peptidase inhibitor, clade A (alpha-1 antiproteinase, antitrypsin),	1.08
74603	Cd200r3	CD200 receptor 3	1.07
69169	Fcmr	Fc fragment of IgM receptor	1.06
75723	Amotl1	angiomotin-like 1	1.06
26364	Adgre5	adhesion G protein-coupled receptor E5	1.05
230576	Ttc22	tetratricopeptide repeat domain 22	1.04
19329	Rab17	RAB17, member RAS oncogene family	1.04
11670	Aldh3a1	aldehyde dehydrogenase family 3, subfamily A1	1.04
231801	Agfg2	ArfGAP with FG repeats 2	1.04
235130	Adamts15	rin-like and metallopeptidase (reprolysin type) with thrombospondin type 1	1.03
66166	S100a14	S100 calcium binding protein A14	1.02
17684	Cited2	/p300-interacting transactivator, with Glu/Asp-rich carboxy-terminal domai	1.02
12334	Capn2	calpain 2	1.02
74007	Btbd11	BTB (POZ) domain containing 11	1.02
102371	Myzap	myocardial zonula adherens protein	1.02
18611	Pea15a	phosphoprotein enriched in astrocytes 15A	1.01
230822	Ncmap	noncompact myelin associated protein	1.01
22325	Vav2	Mus musculus vav 2 oncogene (Vav2), mRNA.	1

13824	Epb41l4a	erythrocyte membrane protein band 4.1 like 4a	1
18140	Uhrf1	ubiquitin-like, containing PHD and RING finger domains, 1	-1
319613	Sybu	syntabulin (syntaxin-interacting)	-1
20309	Cxcl15	chemokine (C-X-C motif) ligand 15	-1.02
17228	Cma1	chymase 1, mast cell	-1.02
193385	Fam65b	family with sequence similarity 65, member B	-1.02
77254	Yif1b	Yip1 interacting factor homolog B (S. cerevisiae)	-1.03
11982	Atp10a	ATPase, class V, type 10A	-1.04
12696	Cirbp	cold inducible RNA binding protein	-1.05
12411	Cbs	cystathionine beta-synthase	-1.05
20510	Slc1a1	family 1 (neuronal/epithelial high affinity glutamate transporter, system Xa	-1.06
53324	Nptx2	neuronal pentraxin 2	-1.07
384009	Glipr2	GLI pathogenesis-related 2	-1.07
12006	Axin2	axin 2	-1.07
71242	Spata24	spermatogenesis associated 24	-1.07
229687		Mus musculus chitinase like protein 2 (Bclp2), mRNA.	-1.08
74448	Arl13a	ADP-ribosylation factor-like 13A	-1.09
72401	Slc43a1	solute carrier family 43, member 1	-1.1
18574	Pde1b	phosphodiesterase 1B, Ca2+-calmodulin dependent	-1.1
319765	Igf2bp2	insulin-like growth factor 2 mRNA binding protein 2	-1.1
268527	Greb1	gene regulated by estrogen in breast cancer protein	-1.1
12583	Cdo1	cysteine dioxygenase 1, cytosolic	-1.1
77254	Yif1b	Yip1 interacting factor homolog B (S. cerevisiae)	-1.1
110197	Dgkg	diacylglycerol kinase, gamma	-1.11
74182	Gpcpd1	glycerophosphocholine phosphodiesterase 1	-1.12
237038	Nox1	NADPH oxidase 1	-1.12
56173	Cldn14	claudin 14	-1.12
76820	Fam49a	family with sequence similarity 49, member A	-1.13
140580	Elmo1	engulfment and cell motility 1	-1.14
236920	Stard8	START domain containing 8	-1.16
214189	Scgn	secretagogin, EF-hand calcium binding protein	-1.16
12978	Csf1r	colony stimulating factor 1 receptor	-1.17
57276	Vsig2	V-set and immunoglobulin domain containing 2	-1.18
13417	Dnah8	dynein, axonemal, heavy chain 8	-1.18
105727	Slc38a1	solute carrier family 38, member 1	-1.18
380686	Cnrip1	cannabinoid receptor interacting protein 1	-1.18
236920	Stard8	START domain containing 8	-1.19
11925	Neurog3	neurogenin 3	-1.2
252838	Tox	thymocyte selection-associated high mobility group box	-1.23
100040736	Foxd2os	forkhead box D2, opposite strand	-1.23
76429	Lhpp	phospholysine phosphohistidine inorganic pyrophosphate phosphatase	-1.23
14587	Gfra3	glial cell line derived neurotrophic factor family receptor alpha 3	-1.25
18166	Npy1r	neuropeptide Y receptor Y1	-1.29
244886	Tmem266	transmembrane protein 266	-1.29
26897	Acot1	acyl-CoA thioesterase 1	-1.29
208213	Tmem132c	transmembrane protein 132C	-1.3
20356	Sema5a	n repeats (type 1 and type 1-like), transmembrane domain (TM) and short cy	-1.3
73914	Irak3	interleukin-1 receptor-associated kinase 3	-1.31
26897	Acot1	acyl-CoA thioesterase 1	-1.31
17173	Ascl2	achaete-scute family bHLH transcription factor 2	-1.32
381524	AI427809	expressed sequence AI427809	-1.32
73296	Rhobtb3	Rho-related BTB domain containing 3	-1.33
17912	Myo1b	myosin IB	-1.34
243277	Adgrd1	adhesion G protein-coupled receptor D1	-1.38
72821	Scn2b	sodium channel, voltage-gated, type II, beta	-1.4
320007	Sidt1	SID1 transmembrane family, member 1	-1.41
12522	Cd83	CD83 antigen	-1.41

20356	Sema5a	n repeats (type 1 and type 1-like), transmembrane domain (TM) and short cy	-1.42
68891	Cd177	CD177 antigen	-1.43
11302	Aatk	apoptosis-associated tyrosine kinase	-1.45
217212	Pyy	peptide YY	-1.46
78252	Nxpe2	neurexophilin and PC-esterase domain family, member 2	-1.49
233066	Syne4	spectrin repeat containing, nuclear envelope family member 4	-1.51
20446	St6galnac2	tyl-neuraminyl-2,3-beta-galactosyl-1,3)-N-acetylgalactosaminide alpha-2,6-	-1.57
14133	Fcna	ficolin A	-1.58
72535	Aldh1b1	aldehyde dehydrogenase 1 family, member B1	-1.59
17173	Ascl2	achaete-scute family bHLH transcription factor 2	-1.62
22371	Vwf	Von Willebrand factor	-1.64
12653	Chgb	chromogranin B	-1.65
19227	Pthlh	parathyroid hormone-like peptide	-1.68
66141	Ifitm3	interferon induced transmembrane protein 3	-1.71
14526	Gcg	glucagon	-1.72
68891	Cd177	CD177 antigen	-1.73
77920	A330102I10Ri	RIKEN cDNA A330102I10 gene	-1.73
381524	AI427809	expressed sequence AI427809	-1.76
12487	Cd28	CD28 antigen	-1.76
14160	Lgr5	leucine rich repeat containing G protein coupled receptor 5	-1.76
67547	Slc39a8	solute carrier family 39 (metal ion transporter), member 8	-1.78
216622	931440F15Ri	RIKEN cDNA 4931440F15 gene	-1.79
18173	Slc11a1	ite carrier family 11 (proton-coupled divalent metal ion transporters), memb	-1.83
23919	Insl5	insulin-like 5	-1.83
18780	Pla2g2a	phospholipase A2, group IIA (platelets, synovial fluid)	-1.85
68713	Ifitm1	interferon induced transmembrane protein 1	-1.93
192187	Stab1	stabilin 1	-1.99
19277	Ptpro	protein tyrosine phosphatase, receptor type, O	-2.01
18947	Pnliprp2	pancreatic lipase-related protein 2	-2.04
270152	Amica1	adhesion molecule, interacts with CXADR antigen 1	-2.05
93695	Gpnmb	glycoprotein (transmembrane) nmb	-2.17
11459	Acta1	actin, alpha 1, skeletal muscle	-2.21
66214	Rgcc	regulator of cell cycle	-2.25
270152	Amica1	adhesion molecule, interacts with CXADR antigen 1	-2.27
16000	Igf1	insulin-like growth factor 1	-2.27
80891	Fcrls	Fc receptor-like S, scavenger receptor	-2.33
93695	Gpnmb	glycoprotein (transmembrane) nmb	-2.38
80891	Fcrls	Fc receptor-like S, scavenger receptor	-2.42
57257	Vav3	vav 3 oncogene	-2.63
109648	Npy	neuropeptide Y	-3.2
11846	Arg1	arginase, liver	-3.3
11846	Arg1	arginase, liver	-3.49
74145	F13a1	coagulation factor XIII, A1 subunit	-3.74

Supplementary Table S7. List of primers

Gene symbol	Primer sequence
Actb	Forward: 5'-GATCTGGCACCACACCTTCT-3'
	Reverse: 5'-GGGGTGTTGAAGGTCTCAAA-3'
Adamdec1	Forward: 5'-GTCCTGGATGTGGGAGAAGA-3'
	Reverse: 5'-TGTGATGTGGTTGGATGCTT-3'
Aldh1a1	Forward: 5'-ACTGGAGTGTGGTGGAGGAC-3'
	Reverse: 5'-CACAGTGATGGCCTTATCCA-3'
Apoe	Forward: 5'-CAGAGCTCCCAAGTCACACA-3'
	Reverse: 5'-AGTCGGTTGCGTAGATCCTC-3'
Asb2	Forward: 5'-AGTCTGTCTCCCGCAATGAC-3'
	Reverse: 5'-ATTCTTGCTGGCCTCGTAGA-3'
Bgn	Forward: 5'-GACAACCGTATCCGCAAAGT-3'
	Reverse: 5'-GTGGTCCAGGTGAAGTTCGT-3'
Cck	Forward: 5'-TACATCCAGCAGGTCCGCAAAG-3'
	Reverse: 5'-CGATGGGTATTCGTAGTCCTCGG-3'
Cd74	Forward: 5'-CACCGAGGCTCCACCTAAAG-3'
	Reverse: 5'-GGGAACACACACCAGCAGTA-3'
Cfd	Forward: 5'-ATGACGACTCTGTGCAGGTG-3'
	Reverse: 5'-ATTGCAAGGGTAGGGGTCTC-3'
Cyp2d26	Forward: 5'-GTGATCGCCTCCCTCATTTA-3'
	Reverse: 5'-GGCTTTATCAGGCAAACCAG-3'
Edn1	Forward: 5'-ACTTCTGCCACCTGGACATC-3'
	Reverse: 5'-GGTGAGCGCACTGACATCTA-3'
Fkbp5	Forward: 5'-GGTTATCAAAGCCTGGGACA-3'
	Reverse: 5'-CGCCTTTCCGTTTGATTCTA-3'
Foxq1	Forward: 5'-CAAGCCCCCATACTCCTACA-3'
	Reverse: 5'-TGACGAAACAGTCGTTGAGC-3'
Fosb	Forward: 5'-TGTCTTCGGTGGACTCCTTC-3'
	Reverse: 5'-GATCCTGGCTGGTTGTGATT-3'
Gapdh	Forward: 5'-AACTTTGGCATTGTGGAAGG-3'
	Reverse: 5'-ATCCACAGTCTTCTGGGTGG-3'
Gcnt1	Forward: 5'-CCGATTGGAGAGTGTGGTTT-3'
	Reverse: 5'-TCCGTTTCCAGGTTGTTTTC-3'
H2-Ab1	Forward: 5'-GTCCTGGTCATGCTGGAGAT-3'
	Reverse: 5'-CTGACTCCTGTGACGGATGA-3'

H2-Ea-ps	Forward: 5'-CTGCCCTCCACAGATGATTT-3'
	Reverse: 5'-GATAATCCCCACAACGATGC-3'
Irs2	Forward: 5'-ACAACCTATCGTGGCACCTC-3'
	Reverse: 5'-GACGGTGGTGGTAGAGGAAA-3'
Ly6a	Forward: 5'-TCTTGTGGCCCTACTGTGTG-3'
	Reverse: 5'-GGCAGATGGGTAAGCAAAGA-3'
Mmp10	Forward: 5'-CAGGTGTGGTGTTCCTGATG-3'
	Reverse: 5'-GGAGAAAGTGAGTGGGGTCA-3'
Nkx2-3	Forward: 5'-GATTCGGGTCTCTGTCCTCA-3'
	Reverse: 5'-AGACCTGAGCTTGCGAGAAG-3'
Oprk1	Forward: 5'-AAGCTTTGGACTTCCGAACA-3'
	Reverse: 5'-GGAAACTGCAAGGAGCATTC-3'
Plvap	Forward: 5'-CATCCTGAGCGAGAAGCAGT-3'
	Reverse: 5'-AGCAGGCTCTCCTTGTCCTT-3'
Reg4	Forward: 5'-GAAACCTGCCTGTGTGGATT-3'
	Reverse: 5'-GCCTGGCTTCACTCTTTGTC-3'
Slc17a4	Forward: 5'-TCCCACTAACTCCCAGGATG-3'
	Reverse: 5'-TTAACAAGCCCAGACCAACC-3'
Slpi	Forward: 5'-CACAATGCCGTACTGACTGG-3'
	Reverse: 5'-GACATTGGGAGGGTTAAGCA-3'
Tgfbr2	Forward: 5'-GGCTTCACTCTGGAAGATGC-3'
	Reverse: 5'-TGACACCCGTCACTTGGATA-3'
Tle4	Forward: 5'-GAGAATGGCCTGGACAAGAC-3'
	Reverse: 5'-GGGTAGGTGCATCAGTTCGT-3'
Ubb	Forward: 5'-ATGTGAAGGCCAAGATCCAG-3'
	Reverse: 5'-TAATAGCCACCCCTCAGACG-3'
Zfp326	Forward: 5'-AACGCCTGCTTATCCTGAAA-3'
	Reverse: 5'-ATCGTCACATTGGCAGGTTT-3'

UPL probes used for qRT-PCR

Gene name	F-primer	R-primer	UPL probe No.
Arg1	gtggggaaagccaatgaag	gcttccaactgccagactgt	20
Ascl2	gagagctaagcccgatgga	aggtccaccaggagtcacc	17
Axin2	gagagtgagcggcagagc	cggctgactcgttctcct	96
Gapdh	aagagggatgctgcccttac	ccattttgtctacgggacga	33
Igfbp7	tgccctccatgaaataccac	ggctgtctgagagcaccttt	110
Krt13	agtcccagctgagcatgaa	gagcccctggatctgttg	53
Krt14	atcgaggacctgaagagcaa	tcgatctgcaggaggacatt	83
Lgr5	gactttaactggagcaaagatctca	cgagtaggttgtaagacaaatctagc	60
Olfm4	ctccgggaggcacttctt	ctgtccacagacccagtgaa	102
Onecut2	gagctcttctttctgacgtatgg	tgtttctttgggggagtctg	5
Wnt7a	cgctgggagagcgtactg	cgataatcgcataggtgaagg	12
Wnt7b	tcatgaaccttcacaacaatga	tggtccagcaagttttggt	92
